# Multi-Omics Prognostic Signatures Based on Lipid Metabolism for Colorectal Cancer

**DOI:** 10.3389/fcell.2021.811957

**Published:** 2022-02-11

**Authors:** YuanLin Sun, Bin Liu, YuJia Chen, YanPeng Xing, Yang Zhang

**Affiliations:** Department of Gastrointestinal Surgery, The First Hospital of Jilin University, Changchun, China

**Keywords:** colorectal cancer, prognosis, multi omics, lipid metabolism, prognostic signature, tumor immune infiltration, tumor microenvironment, somatic mutation

## Abstract

**Background:** The potential biological processes and laws of the biological components in malignant tumors can be understood more systematically and comprehensively through multi-omics analysis. This study elaborately explored the role of lipid metabolism in the prognosis of colorectal cancer (CRC) from the metabonomics and transcriptomics.

**Methods:** We performed K-means unsupervised clustering algorithm and t test to identify the differential lipid metabolites determined by liquid chromatography tandem mass spectrometry (LC-MS/MS) in the serum of 236 CRC patients of the First Hospital of Jilin University (JLUFH). Cox regression analysis was used to identify prognosis-associated lipid metabolites and to construct multi-lipid-metabolite prognostic signature. The composite nomogram composed of independent prognostic factors was utilized to individually predict the outcome of CRC patients. Glycerophospholipid metabolism was the most significant enrichment pathway for lipid metabolites in CRC, whose related hub genes (GMRHGs) were distinguished by gene set variation analysis (GSVA) and weighted gene co-expression network analysis (WGCNA). Cox regression and least absolute shrinkage and selection operator (LASSO) regression analysis were utilized to develop the prognostic signature.

**Results:** Six-lipid-metabolite and five-GMRHG prognostic signatures were developed, indicating favorable survival stratification effects on CRC patients. Using the independent prognostic factors as variables, we established a composite nomogram to individually evaluate the prognosis of CRC patients. The AUCs of one-, three-, and five-year ROC curves were 0.815, 0.815, and 0.805, respectively, showing auspicious prognostic accuracy. Furthermore, we explored the potential relationship between tumor microenvironment (TME) and immune infiltration. Moreover, the mutational frequency of TP53 in the high-risk group was significantly higher than that in the low-risk group (*p* < 0.001), while in the coordinate mutational status of TP53, the overall survival of CRC patients in the high-risk group was significantly lower than that in low-risk group with statistical differences.

**Conclusion:** We identified the significance of lipid metabolism for the prognosis of CRC from the aspects of metabonomics and transcriptomics, which can provide a novel perspective for promoting individualized treatment and revealing the potential molecular biological characteristics of CRC. The composite nomogram including a six-lipid-metabolite prognostic signature is a promising predictor of the prognosis of CRC patients.

## Introduction

The morbidity and mortality of CRC remained stubbornly high in the world, respectively ranking the third and fourth (Gao et al., 2016; Zhang et al., 2017). The progress of CRC was associated with the hereditary factor, age, chronic inflammation, lifestyle such as smoking and drinking, dietary habits, and environmental factors (Aran et al., 2016). At present, the improvement of the prognosis of CRC patients was still fairly limited, mainly because a substantial portion of CRC patients were often asymptomatic in the early stage. However, the mechanism of occurrence, development, and invasion of CRC was still well unclear. Therefore, to find the molecular mechanism and favorable prognostic monitoring index for the occurrence, development and invasion of CRC was the hotspot of the current research.

At present, a large number of studies have shown that abnormal lipid metabolism was closely correlated with the occurrence and development of tumor. The accelerated process of malignant transformation and cancer cell proliferation required more energy, which could induce the dysfunction of lipid metabolism to allow cancer cells to survive (Santos and Schulze, 2012). It has been reported that higher level of plasma total cholesterol (TC) was associated with an increased incidence of CRC (Zalloua et al., 2019), and the absorption of polyunsaturated fatty acids in the diet would moderate the level of TC, thereby reducing the risk of CRC (Muka et al., 2016). In some prospective studies, the level of TC was positively correlated with the occurrence of breast cancer, prostate cancer, and colon cancers. Contrarily, TC level was negatively correlated with the occurrence of hepatocellular cancer, gastric cancer, and lung cancer (Kitahara et al., 2011). The previous studies reported that the levels of low-density lipoprotein (LDL) level in nearly two-fifths of CRC patients presented rising trend, while the high-density lipoprotein (HDL) level in most patients (88.3%) were normal (Liao et al., 2015; Rodriguez-Broadbent et al., 2017). In view of the fact that the dysfunction of lipid metabolism played an important role in genesis and development of tumor, the biological activity of lipid metabolism has always been the key research area screening tumor therapeutic targets. ACOT8 was a kind of peroxisome lipolysis-related enzyme that catalyzed the decomposition of acyl CoA into free fatty acids and CoA for β-oxidation. It has been suggested that ACOT8 was overexpressed in certain kinds of cancers (Ramakrishna et al., 2010; Hung et al., 2014). What is more, downregulated expression of FASN *via* RNAi technique had a significant effect on lipid metabolism inhibition and TG storage of metastatic prostate cancer cells in human lymph nodes (De Schrijver et al., 2003). Since the survival of tumor cells mainly depended on FASN-mediated *de novo* synthesis of fatty acids, FASN was considered as the one of the important targets for human cancer therapy (Lupu and Menendez, 2006).

Metabonomics, an important branch of systematic biology, was mainly utilized to study the changes of metabolites in the dynamic process of metabolism to uncover the metabolic features of life activities. The results obtained from the examination for the metabolites could reflect the pathophysiological state of the organism more accurately and directly. Nowadays, blood and urine were the main research objects, and the accuracies of test results were guaranteed by removing impure metabolites (such as protein or saccharide). The applications of metabonomics (such as LC-MS (Yin et al., 2006), GC-MS (Tian et al., 2016), and CE-MS (DeLaney et al., 2018) and so on) in cancer research were intended to improve the diagnosis and prognosis of cancer, and was employed to distinct potential cancer biomarkers. Tenori et al. found that the recurrence of breast cancer was associated with the decrease of histidine level and the increase of blood glucose level and blood lipid level through the metabonomic analysis for the serum of patients with early breast cancer and metastatic breast cancer (Tenori et al., 2015). Lodi et al. published an NMR-based metabonomic study, which analyzed serum and urine samples from myeloma patients with different stages and metabolic patterns associated with disease progression to identify useful markers for myeloma patients. In this way, patients are grouped based on recurrence or remission (Lodi et al., 2013).

In this study, we firstly utilized LC-MS/MS to determine differential lipid metabolites altas based on the serum samples from 236 CRC patients. The functional enrichment analysis and multi-lipid metabolite prognostic signature analysis revealed the potential biological function and prognostic significance of differential lipid metabolites. Moreover, the prognosis of CRC patients was systematically evaluated by a composite nomogram. In the meantime, aiming at the most significant functional enrichment pathway enriching differential lipid metabolites, namely, glycerophospholipid metabolic pathway, we analyzed the prognostic value of GMRHG from the transcriptional level, and constructed the five-GMRHG prognostic signature. In addition, based on the five-GMRHG prognostic signature, we further expanded the significance of GMRHG in tumor immune infiltration, tumor mutation landscape, and antineoplastic therapy.

## Methods

### Sample Source

In this study, the serum samples were collected from 236 patients (all patients voluntarily signed informed consent forms after being notified full details of the study) hospitalized in the JLUFH from 2008 to 2013, who were pathologically diagnosed with primary CRC, had not been treated with hormonal therapy or chemoradiotherapy, and did not have acute inflammatory reactions. The exclusion criteria were (Zhang et al., 2017) congenital disease, metabolic disease, hematological disease, chronic inflammatory disease, severe cerebro-cardiovascular disease, respiratory disease, liver and renal disease, mental disease (Gao et al., 2016); women who were pregnant or could not rule out possibility of pregnancy and breastfeeding (Aran et al., 2016); alcoholics, drug addicts, long-term use of proton pump inhibitors and hormones or non-steroidal anti-inflammatory drugs (Santos and Schulze, 2012); patients with any acute symptoms or major stress response (such as mental trauma or burns) in the past 2 weeks; and patients who had a cubital venous blood collected on an empty stomach in the early morning. Then, the blood samples were centrifuged for 2 min with 3500 r/min. The upper serum was frozen and stored at −80°C until being used to analyze.

### Metabolic Analysis

Each serum sample taken from refrigerator with temperatures as low as 80°C was dissolved in 500 µl acetonitrile. Then, the serum samples were blended with the vortex device and centrifuged with 14000 r/min at 4°C for 5 min, and the supernatant of each sample was taken for LC-MS analysis, which was conducted using the triple-quadrupole tandem mass spectrometer (AB Sciex TripleTOF 5600, AB Sciex, United States) equipped with the high performance liquid chromatography (HPLC) column (2.1 × 150 mm, 3.5 μm) (Agilent Eclipse Plus C18, California, United States). The mobile phase A (positive ion: 0.1 formic acid and water; anion: water) and mobile phase B (positive ion: 0.1 formic acid and acetonitrile; anion: acetonitrile) were used for gradient elution (mobile phase A: 0–1.5 min: 80–80; 1.5∼7 min: 80–5; 7–10 min: 5–80; 10–11 min: 80–80. Mobile phase B: 0–1.5 min: 20–20; 1.5–7 min: 20–95; 7–10 min: 95–20; 10–11 min: 20–20). The flow rate, sample load, and column temperature were 0.8 ml/min, 20 µl, and 45°C, respectively. The data acquisition software of the Analyst 1.5.1 software package was used to collect and organize the raw metabolic data, and we obtained the spectral peak index, sample name, and peak intensity area with retention time and precise mass-nucleus ratio. Meanwhile, the raw metabolic data matrix of 236 CRC patients was loaded into the MetaboAnalyst 4.0 online tool (https://www.metaboanalyst.ca/faces/home.xhtml) (Chong et al., 2018) to process with normalization, missing value processing, and scaling to acquire the processed metabolic data matrix including mass-charge ratio (m/z), retention time, and peak area.

### Metabonomic Multivariate Statistical Analysis

To further distinguish metabonomic differences in CRC patients, based on the processed metabolic data matrix, we performed K-means unsupervised clustering algorithm to categorize 236 CRC into Kgroup 1 and Kgroup 2, and the procedures were repeated 1,000 times to determine the stabilization of grouping. The “survival R package” was utilized to perform Kaplan-Meier survival analysis for Kgroup 1 and Kgroup 2 to discern their prognostic differences. Principal component analysis (PCA) and partial least square discriminant analysis (PLSDA) were carried out with the MetaboAnalyst 4.0 online tool to ascertain the between-group variance and grouping rationalization. Then, we performed t test in the MetaboAnalyst 4.0 online tool for Kgroup 1 and Kgroup 2 to identify the differential metabolites, which were imported into The Human Metabolome Database (HMDB) (https://hmdb.ca/) to conduct structural identification and search for the differential lipid metabolites. Meanwhile, Kyoto Encyclopedia of Genes and Genomes (KEGG) functional pathway enrichment analysis for the structure-identified differential lipid metabolites was performed based on the MetaboAnalyst 4.0 online tool.

### Multi-Lipid-Metabolite Prognostic Signature Analysis

Integrating the levels of differential lipid metabolites and survival data (survival status and survival time) of 236 CRC patients, we performed univariate Cox regression analysis with “survival R package” to acquire the survival-associated lipid metabolites (*p* < 0.05). Then, multivariate Cox regression analysis with “survival R package” and “survminer R package” was performed on survival-associated lipid metabolite matrix to obtain the multi-lipid metabolite prognostic signature, whose calculation method was listed along these lines: Lipid metabolite score (LMS) = $\Sigma_{x=1}^{n}Coefx\;*\;Levelx$ (The x represented each metabolite for the establishment of the prognostic signature. Each coefficient, which was calculated by the “survival R package”, was the fixed constant corresponding to each metabolite.). Univariate and multivariate independent prognostic analysis involved in clinical characteristics (age, gender, histological type, pathological stage, T staging, N staging, M staging) assessed the prognostic feasibility of the prognostic signature. Meanwhile, we plotted the composite nomogram mediated by independent prognostic factors with “rms R package” to methodically predict the CRC patients’ overall survival. Moreover, clinical correlation analysis further revealed the correlation between the lipid metabolites of the prognostic signature and various clinical characteristics.

### The Acquisition of Glycerophospholipid-Metabolism-Related Hub Genes by WGCNA

The raw CRC RNA expression matrices were obtained from the TCGA database and GEO datasets (GSE17536, GSE38832, and GSE103479). The TCGA Ensemble IDs of RNAs were transformed into the corresponding official symbol IDs retrieved from Ensemble database (https://asia.ensembl.org/index.html). The standard of RNA expression data was converted from FPKM to TPM. Meanwhile, the raw RNA probes of GSE17536, GSE38832, and GSE103479 were pinpointed their symbol IDs in the corresponding “GPL” file. The batch effect was eliminated with “sva R package” software. Differential expressed analysis was conducted for the TCGA gene expression matrix with filter criteria FDR < 0.05, |logFC| > 0.2. GSVA was used to determine the enrichment fractions of gene sets (Hänzelmann et al., 2013). WGCNA was utilized to depict a scale-free co-expression network, indicating that diverse genes with similar expression patterns might have the co-regulation functional pathway (Langfelder and Horvath, 2008). With reference to the above the results of the KEGG functional pathway enrichment analysis for the structure-identified differential lipid metabolites, we concluded that glycerophospholipid metabolism was the pivotal metabolic pathway enriching lipid metabolites. We calculated the TCGA CRC patients’ glycerophospholipid metabolism enrichment fraction of glycerophospholipid metabolism in the KEGG gene set (c2.cp.kegg.v7.4.symbols.gmt) retrieved from the MSigDB (http://www.gsea-msigdb.org/gsea/msigdb) with GSVA. Differential expressed RNA matrix was conducted with WGCNA to construct a co-expression network with the “WGCNA R package” to identify the GMRHGs. In order to enable the constructed co-expression network to be more consistent with the characteristics of the scale-free network, R^2^ = 0.9 was set to determine the soft threshold. Meanwhile, the adjacency matrix was transformed into the topological overlap matrix (TOM) to reduce noise and spurious correlation. Based on the dynamic tree, we distinguished the dynamic shearing module with the minimum number of modular genes set to 30 and the shearing height set to 0.25. The relationships between the module eigengene (ME) gene sets and the clinical traits (age, gender and pathological stage) as well as glycerophospholipid metabolism were evaluated with gene significance (GS) and module membership (MM).

### Multi-GMRHG Prognostic Signature

CRC patients retrieved from the TCGA and GEO (GSE17536, GSE103479 and GSE38832) were respectively categorized into training and test groups. To minimize the impacts of emergencies (such as acute cerebrovascular or cardiovascular disease, massive hemorrhage, and pulmonary embolism, and so forth) on predicting the survival outcome of CRC patients, we excluded the CRC patients with overall survival less than 30 days. Univariate Cox regression analysis and LASSO regression analysis were performed on the training group (TCGA CRC patients) to obtain prognosis-associated GMRHG. After the optimization of LASSO regression analysis with the “glmnet R package” software, we performed multivariate Cox regression analysis on the prognosis-associated GMRHG expression matrix to construct the multi-GMRHG prognostic signature. In the prognostic signature, the glycerophospholipid metabolism score (GMS) was calculated as follows: GMS = $\Sigma_{i=1}^{n}Coefi\;*\;Expi$ (“Expi” symbolized the expression of each GMRHG in the prognostic. “Coefi” denoted the constant corresponding to each GMRHG, and the constant derived from the calculation with “survival R package”). The test group gene expression matrix was substituted into the above formula to obtain the GMS of each sample, which was used to verify the prognostic significance of the prognostic signature.

### Tumor Immune Infiltration and TME Analysis

Tumor immune analysis was utilized to explore relationship between the tumor immune infiltration and the prognostic signature. Through the ssGSEA for the gene expression matrix of TCGA patients in the high- and low-risk groups, single sample gene set enrichment analysis (ssGSEA) (Barbie et al., 2009), a single-sample function enrichment analysis for a particular gene set, defined an enrichment score indicating the absolute enrichment of the particular gene set in each sample. In this study, we conducted ssGSEA with the “GSVA R package” to calculate the relative abundances of tumor immunocytes and immune functions of each TCGA CRC sample (Angelova et al., 2015). Moreover, we performed the ESTIMATE algorithm to calculate the Immune score and Stromal score to quantify the immune and stromal components in tumor microenvironment (TME), and the sum of both was ESTIMATE score, which was utilized to evaluate the tumor infiltrations in TME. Tumor Immune Dysfunction and Exclusion (TIDE) algorithm was utilized to predict the anti-PD-1 and anti-CTLA4 immunotherapeutic response of CRC patients (Jiang et al., 2018). With the TIDE score escalating, the probability of immune escape escalated and the immunotherapeutic response worsened.

### Somatic Mutation Landscape in the Prognostic Signature

The somatic mutation data retrieved from the TCGA “Masked Somatic Mutation” data type was summarized with the “maftools R package” to depict the genetic mutational landscape of CRC patients and of the characteristic genes of mismatch repair (MMR) system, namely, MLH1, MSH2, MSH6, and PMS2.

### Gene Set Enrichment Analysis for the Prognostic Signature

GSEA, the connectivity analysis performed on the specific functional phenotypes for the whole gene chip expression profiling (Subramanian et al., 2005), was utilized to evaluate the enrichment of the gene expression matrices of high- and low-risk groups in the KEGG gene sets and hallmark gene set (NOM *p* < 0.05, FDR *q* < 0.25), thereby hierarchically identifying the specific biological function of CRC patients in high- and low-risk groups.

## Results

### Metabonomics Pattern Recognition Analysis

**FIGURE 1:**
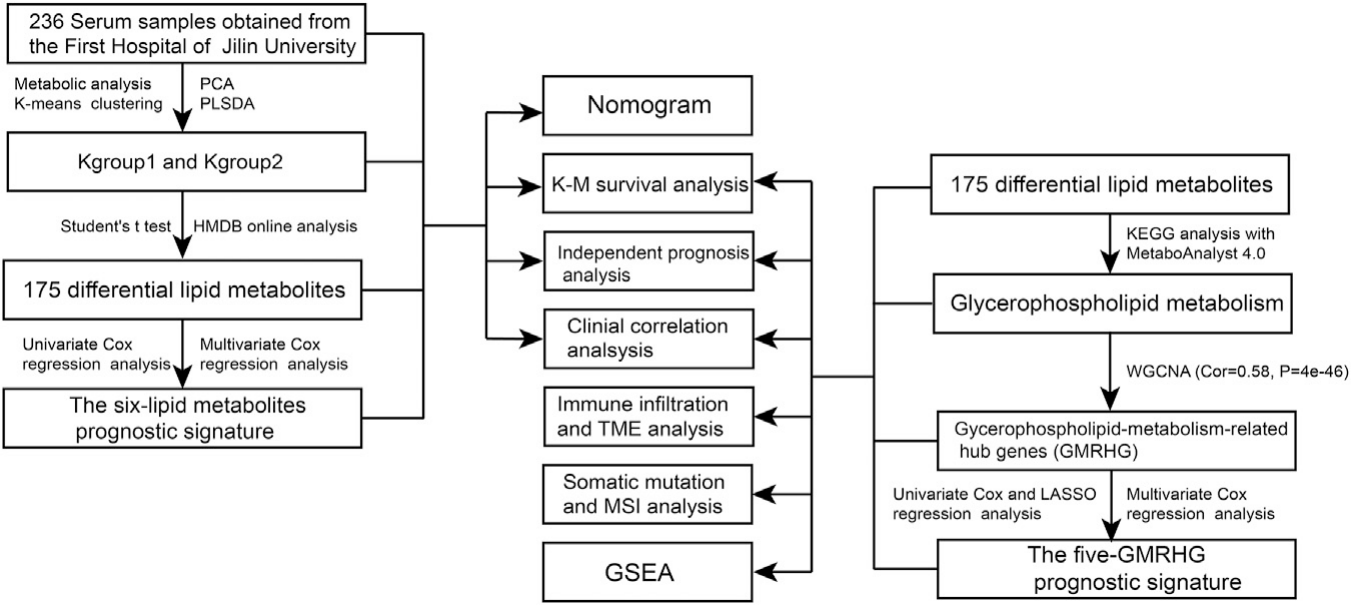
The flow chart of the study.

**FIGURE 2:**
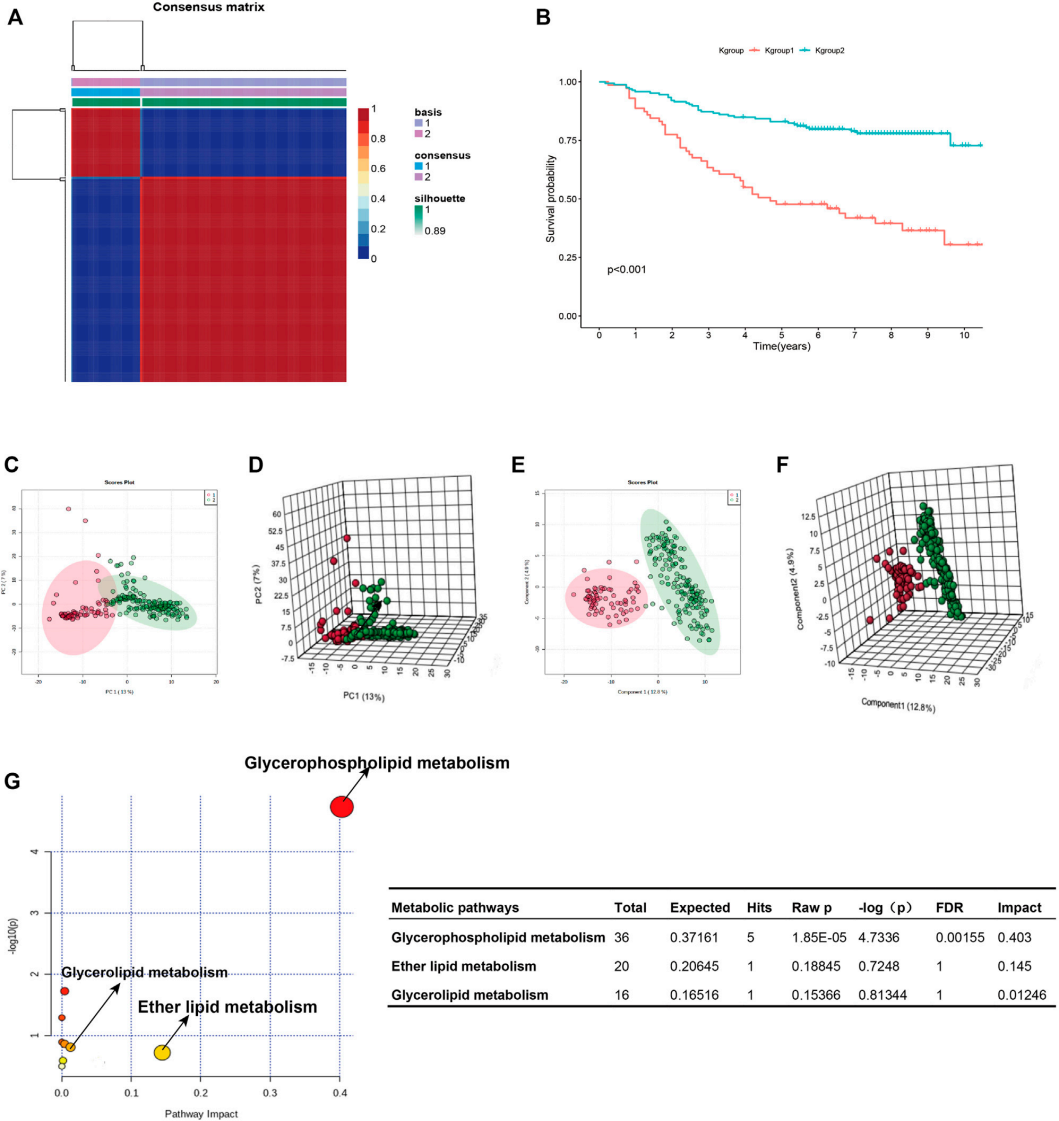
K-group1 and K-group2 are independent with each other. **(A)**: K-means clustering heatmap of K-group1 and K-group2. **(B)**: The K-M survival curves indicated that the overall survival of JLUFH CRC patients in K-group 1 was lower than that in K-group2. **(C,D)**: Two-dimensional and three-dimensional PCA plots for K-group1 and K-group2. **(E,F)**: Two-dimensional and three-dimensional PLSDA plots for K-group1 and K-group2. **(G)**: The KEGG functional enrichment analysis for differential lipid metabolites.

Figure 1 shows the research idea of the study. Table 1 shows the clinical statistics of CRC patients. Totally, we included 236 CRC patients with their clinical characteristics, including age, gender, histological type, pathological stage, T staging, N staging, and M staging. Moreover, we performed PCA and PLSDA to investigate the spatial distribution of metabolites in the subgroups (Kgroup 1 and Kgroup 2) completed with K-means unsupervised clustering algorithm (Figure 2A and Supplementary Table S1). K-M survival analysis (Figure 2B) indicated that the overall survival of CRC patients in Kgroup 2 was higher than that in Kgroup 1 (*p* < 0.001). Figures 2C,D show the results of PCA for Kgroup 1 and Kgroup 2, indicating that there were significant individual differences between two subgroups. Furthermore, in order to eliminate random deviations and deviations within the group, we performed PLSDA for Kgroup 1 and Kgroup 2 to further validate the individual differences between two subgroups (Figures 2E,F).

**TABLE 1:** The overview of JLUFH CRC patients’ clinical characteristics.

Characteristics	Variates	Amounts (Percentage%)
Age	<=65	168 (71.19)
	>65	68 (28.81)
Gender	Female	106 (44.92)
	Male	130 (55.08)
Histological type	Adenocarcinoma	191 (80.93)
	Mucinous adenocarcinoma	45 (19.07)
T staging	T1-2	35 (14.83)
	T3-4	201 (85.17)
N staging	N0	124 (52.54)
	N1-2	112 (47.46)
M staging	M0	225 (95.34)
	M1	11 (4.66)
Pathological stage	Stage I–II	124 (52.54)
	Stage III–IV	112 (47.46)

### The Biological Functions of Differential Metabolites

T test was employed to distinguish the differential 357 metabolites (FDR < 0.05), which were then conducted identifications of specific components on HMDB (https://hmdb.ca/). We confirmed 175 differential lipid metabolites (Supplementary Table S2), notably, most of which were fatty acid and glycophospholipin such as glycolipid products. KEGG pathway enrichment analysis on the MetaboAnalyst 4.0 online tool for the 175 differential lipid metabolites revealed that the differential lipid metabolites uppermost enriched in the glycerophospholipid metabolism (FDR = 0.0016, Impact = 0.403) (Figure 2G).

### The Construction of the Six-Lipid Metabolite Prognostic Signature

**FIGURE 3:**
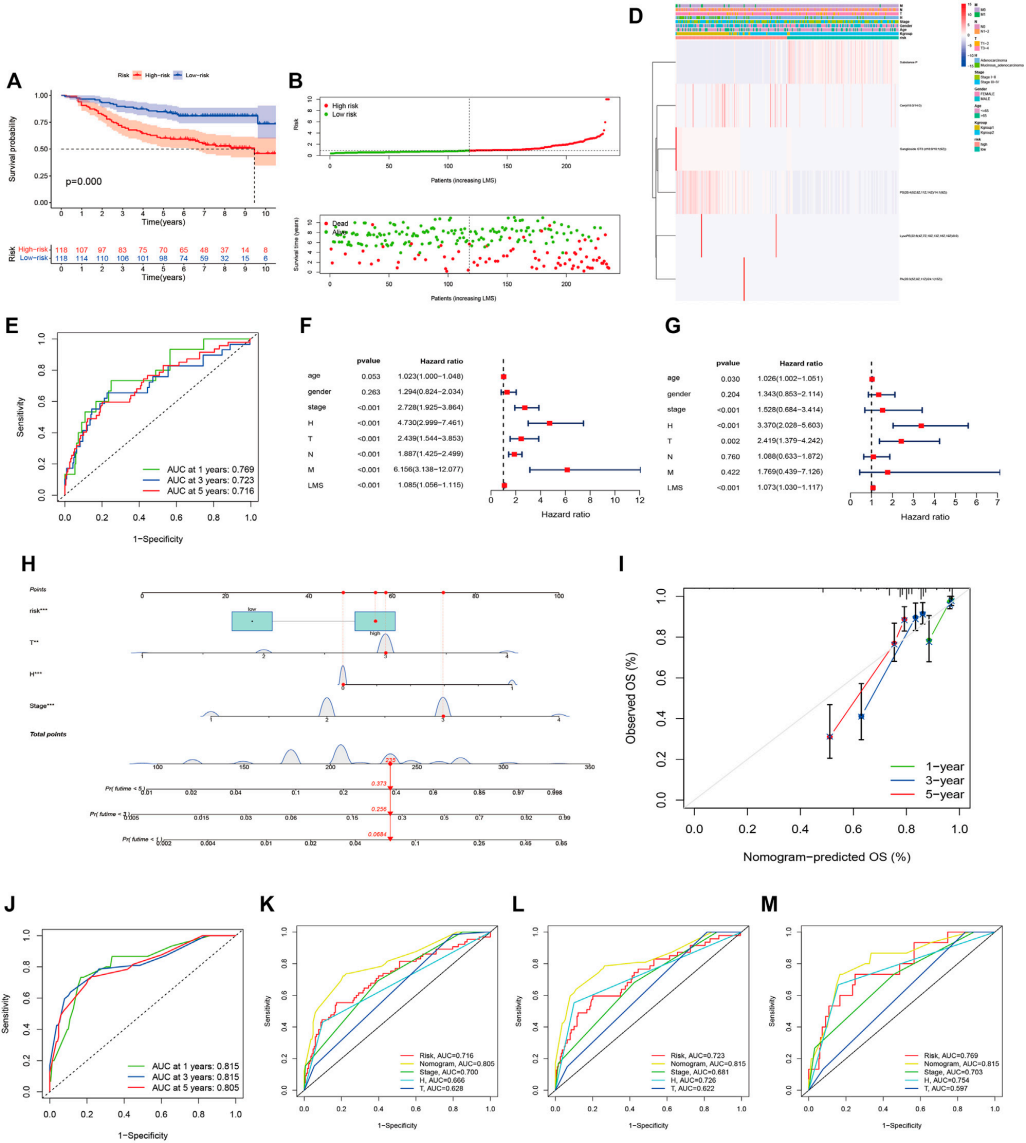
The six-lipid-metabolite prognostic signature is the robust independent prognostic factor. **(A)**: The K-M survival curves based on the six-lipid metabolite prognostic signature. **(B)**: The scatter plot showing the correlation between the JLUFH CRC patients’ survival status and LMS. **(C)**: The dot plot representing the distributions of JLUFH CRC patients’ LMS in the high- and low-risk groups. **(D)**: The clustering heatmap exhibiting the level variation tendencies of the six lipid metabolites of the prognostic signature in various clinical characteristics, K-groups (K-group1 and K-group2) and risk grouping (High- and low-risk groups). **(E)**: The one-, three-, and five-year ROC curves of the six-lipid-metabolite prognostic signature. **(F,G)**: Univariate and multivariate independent prognostic analysis based on the six-lipid-metabolite prognostic signature, demonstrating that pathological stage, histological type and LMS could be deemed as the independent prognostic factors. **(H)**: The composite nomogram consisted of independent prognostic factors. **(I)**: The one-, three-, and five-year calibration curves of the composite nomogram. **(J):** The one-, three-, and five-year ROC curves of the composite nomogram. **(K–M)**: The one-, three-, and five-year multi-index ROC curves of the composite nomogram.

Univariate Cox regression analysis conducted with the “survival R package” was utilized to investigate the survival-associated lipid metabolites (*p* < 0.05) and 14 survival-associated lipid metabolites were confirmed (Table 2). Then, we performed multivariate Cox regression analysis for the 14 survival-associated lipid metabolite to establish the prognostic signature based on six lipid metabolites (Table 3) including five high-risk lipid metabolites (HR > 1) (Cer(d18:0/14:0), Ganglioside GT3(d18:0/18:1(9Z), LysoPE(22:6(4Z,7Z,10Z,13Z,16Z, 19Z)/0:0), PA(20:3(5Z,8Z, 11Z)/24:1(15Z)), PS (20:4(5Z,8Z,11Z, 14Z)/14:1(9Z), and one low-risk lipid metabolites (HR < 1) (Substance P). LMS = 0.233* Level_Cer(d18:0/14:0)_ + 0.303 * Level_LysoPE[22:6(4Z, 7Z, 10Z, 13Z,16Z, 19Z)/0:0]_ + 0.298 * Level_PS (20:4(5Z, 8Z, 11Z, 14Z)/14:1(9Z))_ + 0.968 * Level_PA [20:3(5Z, 8Z, 11Z)/24:1(15Z)]_ + 1.079 * Level_Ganglioside GT3[d18:0/18:1(9Z)]_ + (−0.179) * Level_Substance P_. Patients with an LMS greater than or equal to the median LMS (0.875) were categorized into the high-risk group, and those less than the median LMS (0.875) were classified into the low-risk group. The K-M survival curve (Figure 3A) for CRC patients in the high- and low-risk groups illustrated that the overall survival of the CRC patients in the low-risk group was higher than that in the high-risk group with significant statistical difference (*p* = 0.000). As shown in Figure 3B, the scatter plots showed that the overall survival of CRC patients with higher LMS was lower than that of those with lower LMS. The LMS curve (Figure 3C) showed the distributions of CRC patients with the LMS increasing. The heatmap (Figure 3D) showed the distributions and variations of six metabolite levels based on the prognostic signature. With the increase of LMS, the levels of five high-risk metabolites increased, and the level of the low-risk metabolites showed a decreasing trend. Furthermore, the AUCs of the ROC curves (Figure 3E) to predict the patients’ one-, three-, and five-year overall survival was 0.769, 0.711, and 0.723, respectively. Combined with the LMS and clinical characteristics (age, gender, pathological stage, histological type, T staging, N staging, and M staging), univariate and multivariate independent prognostic analyses (Figures 3F,G) were conducted to examine the prognostic feasibility of the prognostic signature, and the LMS could be an independent prognostic factor to predict the patients’ prognosis. With the integration of the prognostic signature and independent prognostic factors (histological type (adenocarcinoma and mucinous adenocarcinoma), pathological stage, T staging), we constructed the composite nomogram (Figure 3H) to individually predict the CRC patients’ overall survival. The sum of the corresponding scores of variates in the composite nomogram identified the less than one-, three-, and five-year overall survival probability. The one-, three-, and five-year calibration curves also verified the promising fitting and stability of the composite nomogram (Figure 3I). The one-, three-, and five-year AUCs of ROCs based on the nomogram was 0.815, 0.815, and 0.805, respectively, showing favorable accuracy in predicting patients’ prognosis (Figure 3J). The one-, three-, and five-year AUCs of multi-index ROC curves (Figures 3K–M) verified the enhanced prognostic accuracy of the composite nomogram.

**TABLE 2:** Univariate Cox regression analysis for the differential lipid metabolites.

Name	HMDB	HR	HR.95L	HR.95H	*p*-value
PA[16:0/14:1(9Z)]	HMDB0114834	1.341	1.075	1.673	0.019
Substance P	HMDB0001897	0.699	0.554	0.883	0.006
Tridecanoylglycine	HMDB0013317	1.251	1.046	1.497	0.03
PA[14:1(9Z)/22:6(4Z, 7Z, 10Z, 13Z, 16Z, 19Z)]	HMDB0114809	0.75	0.591	0.951	0.036
PGP(a-13:0/a-13:0)	HMDB0116512	1.352	1.116	1.637	0.005
PS(20:4(5Z, 8Z, 11Z, 4Z)/14:1(9Z))	HMDB0012430	1.589	1.332	1.894	0
PA(20:3(5Z, 8Z, 11Z)/24:1(15Z))	HMDB0115143	2.987	1.437	6.209	0.008
PS[18:0/20:4(8Z, 11Z, 14Z, 17Z)]	HMDB0010165	1.194	1.048	1.361	0.016
PS[DiMe(9,3)/MonoMe(11,3)]	HMDB0061584	0.726	0.579	0.909	0.012
Ganglioside GD2(d18:0/16:0)	HMDB0011839	0.744	0.592	0.935	0.023
Ganglioside GT3(d18:0/18:1(9Z))	HMDB0012057	1.309	1.144	1.497	0
Cer(d18:0/14:0)	HMDB0011759	1.32	1.087	1.603	0.011
LysoPC(18:0)	HMDB0010384	1.166	1.03	1.321	0.032
LysoPE[22:6(4Z,7Z,10Z,13Z,16Z,19Z)/0:0]	HMDB0011526	1.168	1.051	1.296	0.008

**TABLE 3:** Multivariate Cox regression analysis for the survival-associated lipid metabolites.

Name	Coef	HR	HR.95L	HR.95H	*p* value
Cer(d18:0/14:0)	0.23250734	1.2617597	1.027315	1.5497072	0.026629
LysoPE[22:6(4Z,7Z,10Z,13Z,16Z,19Z)/0:0]	0.30315863	1.3541293	1.084041	1.6915098	0.0075644
PS[20:4(5Z,8Z,11Z,14Z)/14:1(9Z)]	0.2984225	1.3477311	1.0988127	1.653038	0.0041774
PA[20:3(5Z,8Z,11Z)/24:1(15Z)]	0.96788033	2.6323588	1.2307888	5.6299772	0.0125841
Ganglioside GT3[d18:0/18:1(9Z)]	1.07881366	2.9411882	1.7988826	4.8088675	1.70E-05
Substance P	−0.1788966	0.8361924	0.6503613	1.0751218	0.1629861

### Identification of GMRHGs With WGCNA

**FIGURE 4:**
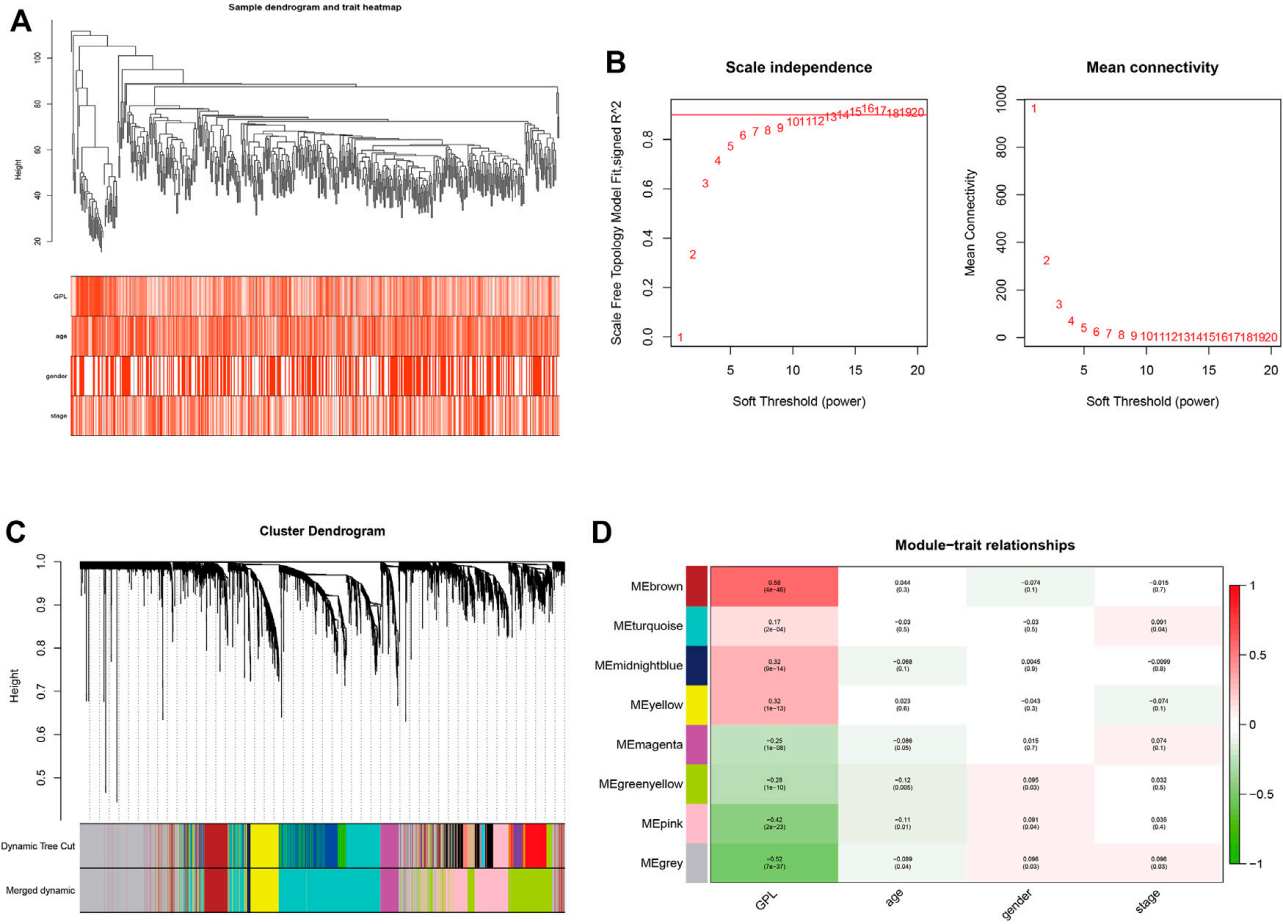
The identification of GMRHGs with WGCNA. **(A)**: Hierarchical clustering dendrogram of TCGA CRC patients. **(B)**: The selection of soft threshold. **(C)**: The dynamic tree cut dendrogram for TCGA CRC patients. **(D)**: The correlation between gene module and clinical traits.

In order to ensure the tumor-specific gene selection, we used differential expressed gene matrix of TCGA CRC patients for WGCNA. Integration of 4432 DEG expression data (Supplementary Figure S1) and age, gender, and pathological stage was used for the input files of WGCNA. Hierarchical clustering (Figure 4A) and Pearson correlation analysis were carried out to construct WGCNA network. Soft threshold β = 13 (Figure 4B) assessed the optimal power value adjacency matrix transforming to TOM. Through the identification of dynamic tree cutting modules and the merge of similar modules (Figure 4C), 8 gene modules (MEbrown, MEturquoise, MEmidnightblue, MEyellow, MEmagenta, MEgreenyellow, MEpink, MEgrey) were identified (Figure 4D), among which the MEbrown module containing 342 DEGs was significantly correlated with glycerophospholipid metabolism (Cor = 0.58, *p* = 4e-46) (Supplementary Table S3). A total of 342 DEGs of MEbrown module were deemed as the GMRHG for further analysis.

### The Construction of the Five-GMRHG Prognostic Signature

**FIGURE 5:**
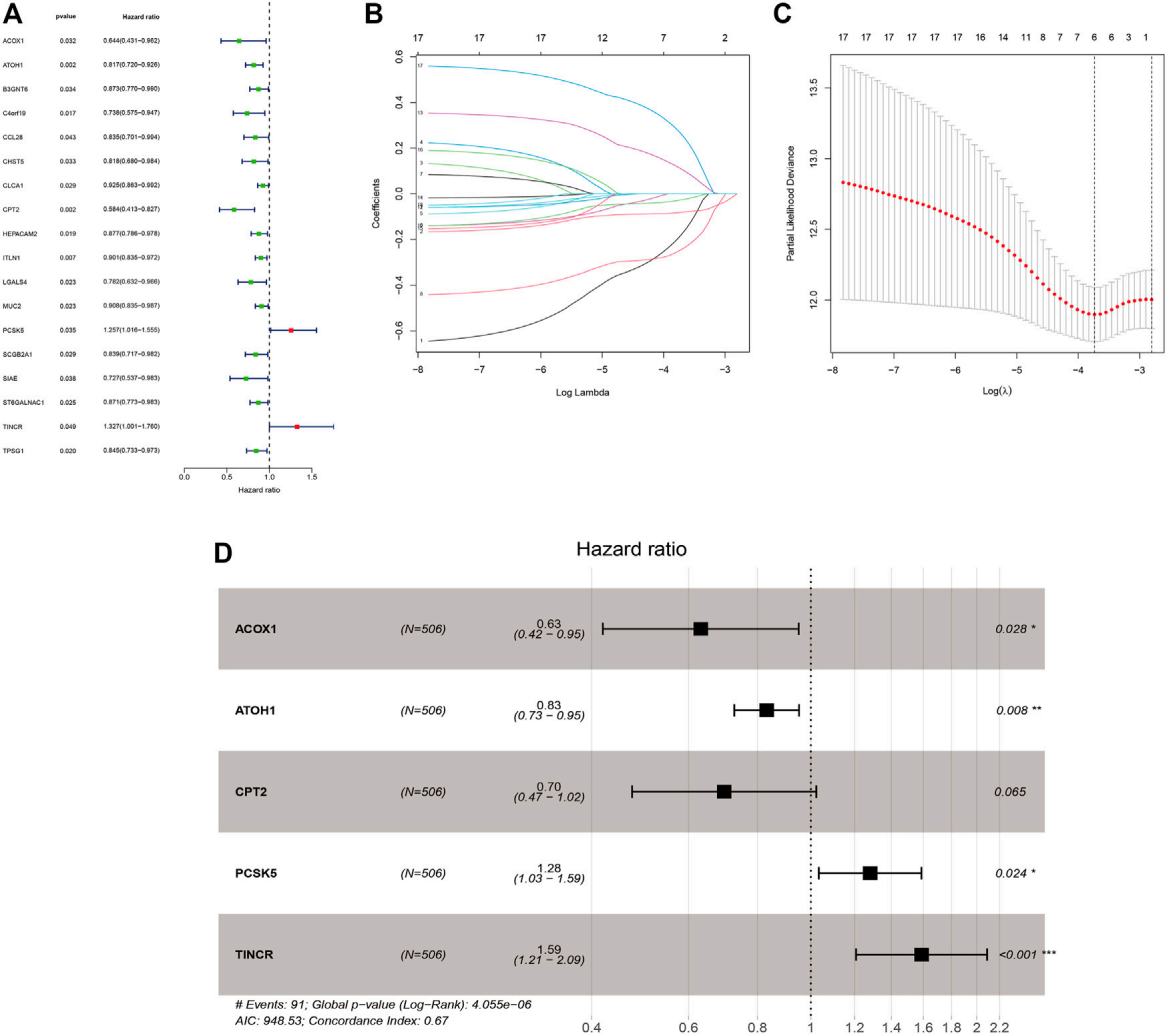
The construction of the five-GMRHG prognostic signature. **(A**): 18 prognosis-associated GMRHGs screened by univariate Cox regression analysis. **(B,C)**: The LASSO regression analysis for the prognosis-associated GMRHGs. **(D)**: Five prognosis-associated GMRHGs screened by multivariate Cox regression analysis.

**FIGURE 6:**
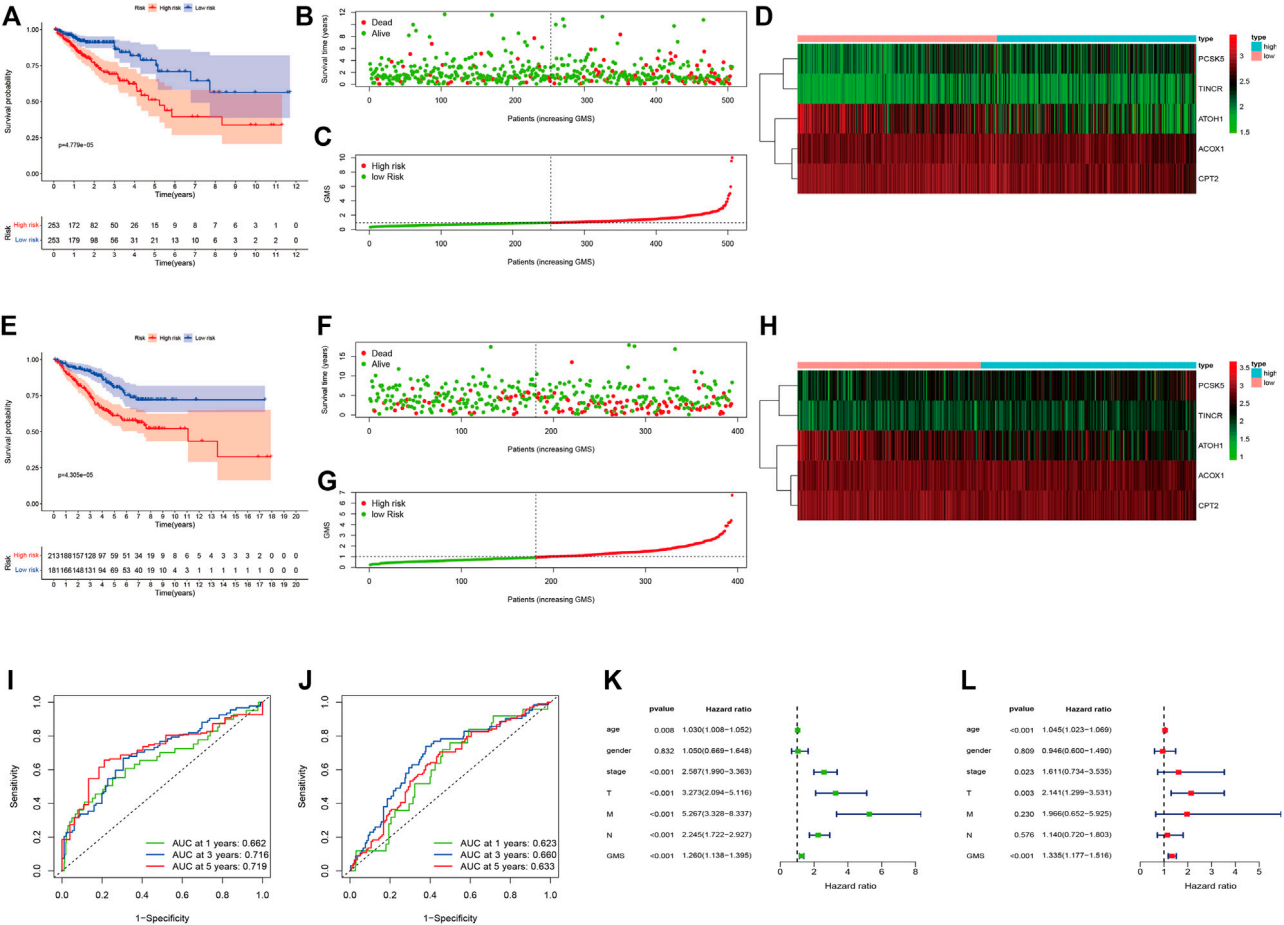
CRC patients stratified according to the five-GMRHG prognostic signature. **(A–D)**: The survival plots for the TCGA CRC patients respectively illustrating the overall survival difference, the distribution of GMS, the survival status of CRC patients, and the expression variations of five GMRHGs in the high- and low-risk groups. **(E)–(H)**: The survival plots for the GEO CRC patients (GSE17536, GSE38832 and GSE103479) respectively illustrating the overall survival difference, the distribution of GMS, the survival status of CRC patients, and the expression variation of five GMRHGs in the high- and low-risk groups. **(I,J)**: The one-, three-, and five-year ROC curves of the five-GMRHG prognostic signature based on the training and test group. **(K,L)**: The univariate and multivariate independent prognostic analysis.

Supplementary Table S4 displayed the clinical statistics of 877 CRC patients retrieved from the TCGA and GEO datasets (GSE17536, GSE38832, and GSE103479). Univariate Cox regression analysis (Figure 5A), LASSO regression analysis (Figures 5B,C), and multivariate Cox regression analysis (Figure 5D) were performed on 342 GMRHGs expression data to construct the five-GMRHG (ACOX1, ATOH1, CPT2, PCSK5, and TINCR) prognostic signature. GMS = (−0.460) * Exp_ACOX1_ +(−0.184) * Exp_ATOH1_ + (−0.362) * Exp_CPT2_ + 0.248 * Exp_PCSK5_ + 0.463 * Exp_TINCR_. CRC patients with a GMS greater than or equal to the median GMS (0.939) would be assigned into the high-risk group, and those less than the median GMS would be assigned into the low-risk group. K-M survival curve (Figure 6A) for the training and test group (Figure 6E) illustrated that there were significant differences of the overall survival between low-risk group and high-risk group (*p* = 4.779e−05 and *p* = 4.305e−05). Scatter plots for the training (Figure 6B) and test group (Figure 6F) indicated that the overall survival of patients with higher GMS was lower than that of patients with lower GMS. GMS curves for the training (Figure 6C) and test group (Figure 6G) showed the distributions of GMS, with the GMS increasing. The heatmaps for the training (Figure 6D) and test group (Figure 6H) showed the expression trends of five GMRHGs of the prognostic signature. With the increase of GMRHG, the expression of two high-risk GMRHGs (PCSK5 and TINCR) would be increased, while the expression of three low-risk GMRHGs (ATOH1 and ACOX1 and CPT2) would be decreased. The one-, three-, and five-year AUCs of time-dependent ROCs in the training (Figure 6I) and test group (Figure 6J) were 0.662, 0.716, 0.719 and 0.623, 0.660, 0.633, respectively. As shown in Figures 6K, L, the results of univariate and multivariate independent prognostic analysis indicated that GMS could be deemed as independent prognostic factors. The time-dependent ROC verified the preferable precision of the five-GMRHG prognostic signature to predict CRC patients’ prognosis.

### Clinical Correlation Analysis

**FIGURE 7:**
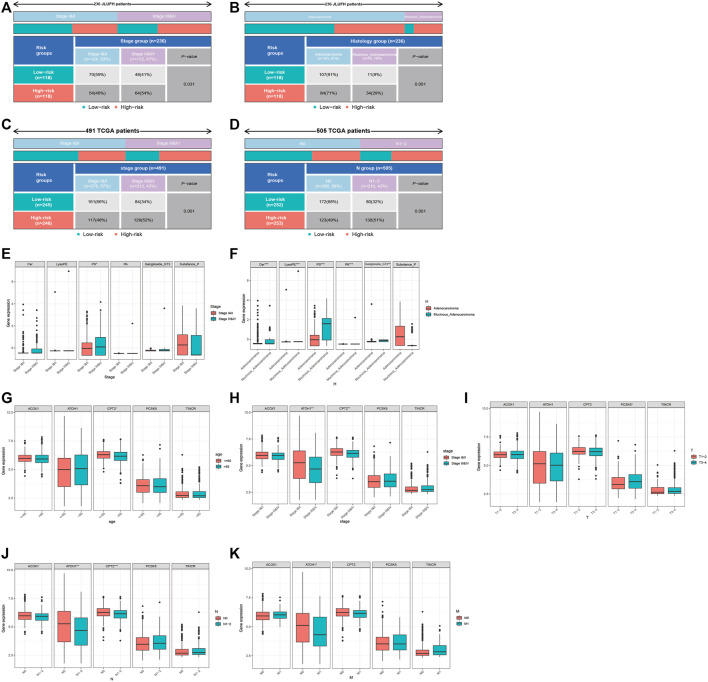
Clinical correlation analysis for the JLUFH and TCGA CRC patients. **(A,B)**: The distributions of pathological stage and histological type of JLUFH CRC patients in the high- and low-risk groups. **(C,D)**: The distributions of pathological stage and N staging of JLUFH CRC patients in the high- and low-risk groups. **(E,F)**: The differences of the levels of six lipid metabolites in the JLUFH CRC patients with stage I and II, stage III and IV and adenocarcinoma, and mucinous adenocarcinoma. **(G–K)**: The differences of the levels of five GMRHGs in the TCGA CRC patients with various clinical characteristics (age, pathological stage, T staging, N staging, and M staging).

We used the Chi-square test to compare the distributions of clinical characteristics (age, gender, histological type, pathological stage, T staging, N staging, M staging) based on JLUFH CRC patients and TCGA CRC patients (age, gender, pathological stage, T staging, N staging, M staging) in the high- and low-risk groups. For the JLUFH CRC patients, we found that CRC patients with advanced pathological stage (stage III and IV) and mucinous adenocarcinomas were mainly distributed in the high-risk group with significant statistical difference (*p* = 0.031 and *p* = 0.001) (Figures 7A,B). For the TCGA CRC patients, we found that TCGA CRC patients with advanced pathological stage (stage Ⅲ and Ⅳ) and N staging (N1-2) were mostly distributed in the high-risk group (Figures 7C,D). Clinical correlation analysis with Wilcoxon ranking test was utilized to further explore the intra-group differences of six-lipid-metabolites level and five-GMRHG expression in clinical characteristics based on the JLUFH CRC patients and the TCGA CRC patients. “*”, “**”, and “***” respectively represented statistical difference, highly statistical difference, and significant statistical difference. It was found that the level of high-risk lipid metabolite, PS [20:4 (5Z, 8Z, 11Z, 14Z)/14:1 (9Z)], was higher in the JLUFH CRC patients in the advanced-pathological-stage group (Figure 7E), and the levels of five high-risk lipid metabolites (Cer(d18:0/14:0), Ganglioside GT3(d18:0/18:1(9Z), LysoPE[22: 6 (4Z, 7Z, 10Z, 13Z, 16Z, 19Z)/0: 0], PA[20:3 (5Z,8Z, 11Z)/24:1 (15Z)], PS[20:4 (5Z, 8Z, 11Z, 14Z)/14:1 (9Z)] in the mucinous-adenocarcinoma group were significantly higher than those in the adenocarcinoma group (Figure 7F). The expression of CPT2 was higher in the TCGA CRC patients no more than 65 years old compared with that in CRC patients greater than 65 years old (Figure 7G). Furthermore, the expressions of ATOH1 and CPT2 presented statistical differences in terms of pathological stage. The expression of ATOH1 and CPT2 was higher in stage Ⅰ and Ⅱ than that in stage Ⅲ and Ⅳ (Figure 7H). In T staging, the expression of PCSK5 in T3-4 was higher than that in T1-2 (Figure 7I). Moreover, in N staging, with the increase of N staging, the expression of ATOH1 and CPT2 displayed downward tendencies (Figure 7J). For M staging, the expression of ATOH1 was higher in M0 than in M1 with statical differences (Figure 7K).

### Characteristics of Tumor Immune Infiltration and TME Based on the Prognostic Signature

**FIGURE 8:**
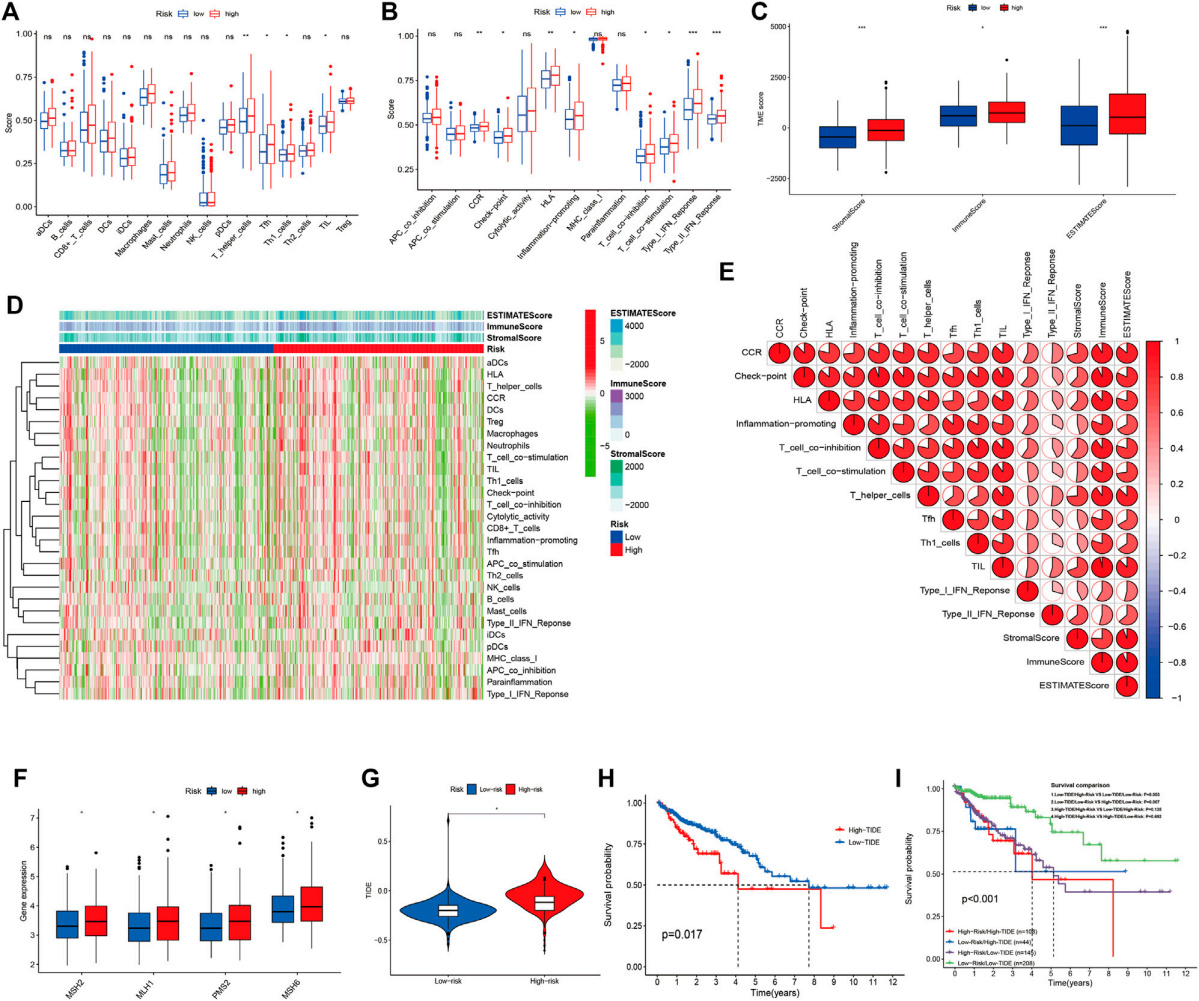
The correlation between the immune infiltrations and TME. **(A,B)**: The differences of relative abundances of immune components in the high- and low-risk groups. **(C)**: The differences of Immune score, Stromal score and ESTIMATE score in the high- and low-risk groups. **(D)**: The distributional correlation between the immune components and TME scores. The red and green strips respectively represent the up-regulated and downregulated immune components. **(E)**: The co-expression relationship among the differential immune components (T_helper_cells, Tfh, Th1_cells, TIL, CCR, check-point, HLA, inflammation-promoting, T_cell_co-inhibition, T_cell_co-stimulation, type_Ⅰ_IFN_Response, and type_Ⅱ_IFN_Response) and TME scores. **(F)**: The expression differences of five immune checkpoints (CTLA4, PDCD1, TIGIT, CD274, and HAVCR2) in the high- and low-risk groups. **(G)**: The difference of TIDE score in the high- and low-risk groups. **(H)**: The K-M survival curves indicating the overall survival difference between the TCGA CRC patients with high-TIDE score and low-TIDE score. **(I)**: The K-M survival curves stratified by the five-GMRHG prognostic signature and TIDE score. The overall survival of TCGA CRC patients with Low-TIDE/High-Risk was lower than that with Low-TIDE/Low-Risk with statistical differences (*p* = 0.003). The overall survival of TCGA CRC patients with Low-TIDE/Low-Risk was lower than that with High-TIDE/Low-risk with statistical differences (*p* = 0.007). Moreover, there were no statistical differences of the overall survival between TCGA CRC patients with High-TIDE/High-Risk VS TCGA CRC patients with Low-TIDE/High-Risk (*p* = 0.135) and TCGA CRC patients with High-TIDE/High-Risk VS TCGA CRC patients with High-TIDE/Low-Risk (*p* = 0.692).

We calculated the relative abundances of 16 kinds of immunocytes and 13 kinds of immune functions with the “GSVA R package”. The box plot (Figures 8A,B) iconically illustrated that the relative abundances of T_helper_cells, Tfh, Th1_cells, TIL, CCR, check-point, HLA, inflammation-promoting, T_cell_co-inhibition, T_cell_co-stimulation, Type_Ⅰ_IFN_Response, and Type_Ⅱ_IFN_Response in the high-risk group were higher than that in the low-risk group with statistical differences (*p* < 0.05). Meanwhile, we applied Spearman correlation analysis to further validate the linear relationship between the prognostic signature and tumor immune infiltration. As shown in Supplementary Figures S2A–2L, the GMS was respectively positively correlated with the relative abundances of T_helper_cells, Tfh, Th1_cells, TIL, CCR, check-point, HLA, inflammation-promoting, T_cell_co-inhibition, T_cell_co-stimulation, Type_Ⅰ_IFN_Response, and Type_Ⅱ_IFN_Response. In this study, we further uncovered the underlying correlations between the TME and tumor immune infiltrations. Figure 8C shows that Immune score, Stromal score, and ESTIMATE score were higher in the high-risk group than that in the low-risk group with statistical differences (*p* < 0.05). Supplementary Figures S2M–2O demonstrated that GMS was respectively linearly positively correlated with Stromal score, Immune score, and ESTIMATE score. The heatmap (Figure 8D) systematically depicted the distributional relationships between the Stromal score, Immune score, ESTIMATE score, and the relative abundances of tumor immunocytes and immune functions in the high- and low-risk groups. The distributions of the Stromal score, Immune score, and ESTIMATE score were collaborative with the distributions of the relative abundances of T_helper_cells, Tfh, Th1_cells, TIL, CCR, check-point, HLA, inflammation-promoting, T_cell_co-inhibition, T_cell_co-stimulation, Type_Ⅰ_IFN_Response, and Type_Ⅱ_IFN_Response in the high- and low-risk groups. Moreover, as shown in the Figure 8E, we found that the Stromal score, Immune score, and ESTIMATE score were all statistically positively correlated with the relative abundances of T_helper_cells, Tfh, Th1_cells, TIL, CCR, check-point, HLA, inflammation-promoting, T_cell_co-inhibition, T_cell_co-stimulation, Type_Ⅰ_IFN_Response, and Type_Ⅱ_IFN_Response in the high- and low-risk groups. The results of the comparisons between the expression of immune checkpoints in the high- and low-risk groups indicated that the expression of CTLA4, PDCD1, TIGIT, CD274, and HAVCR2 was higher in the high-risk group than in the low-risk group (Figure 8F). Spearman correlation analysis (Supplementary Figures S2P–2T) also validated the positive linear correlations between the expression of immune checkpoints and GMS. Furthermore, immunotherapeutic response analysis conducted with the TIDE algorithm was performed on the CRC patients in the high- and low-risk groups. As shown in the Figure 8G, the TIDE score in the low-risk group was lower than that in the high-risk group, indicating that samples in the low-risk group were more susceptive to anti-PD1 and anti-CTLA4 immunotherapy compared with samples in the high-risk group. CRC patients with lower TIDE scores were associated with favorable overall survival compared with CRC patients with higher TIDE scores (*p* = 0.017) (Figure 8H). Moreover, the results of stratified survival analysis for CRC patients in the high- and low-risk groups showed that CRC patients in the low-risk group with lower TIDE scores were related with better performance of overall survival (Figure 8I).

### Somatic Mutation and MSI Overview

**FIGURE 9:**
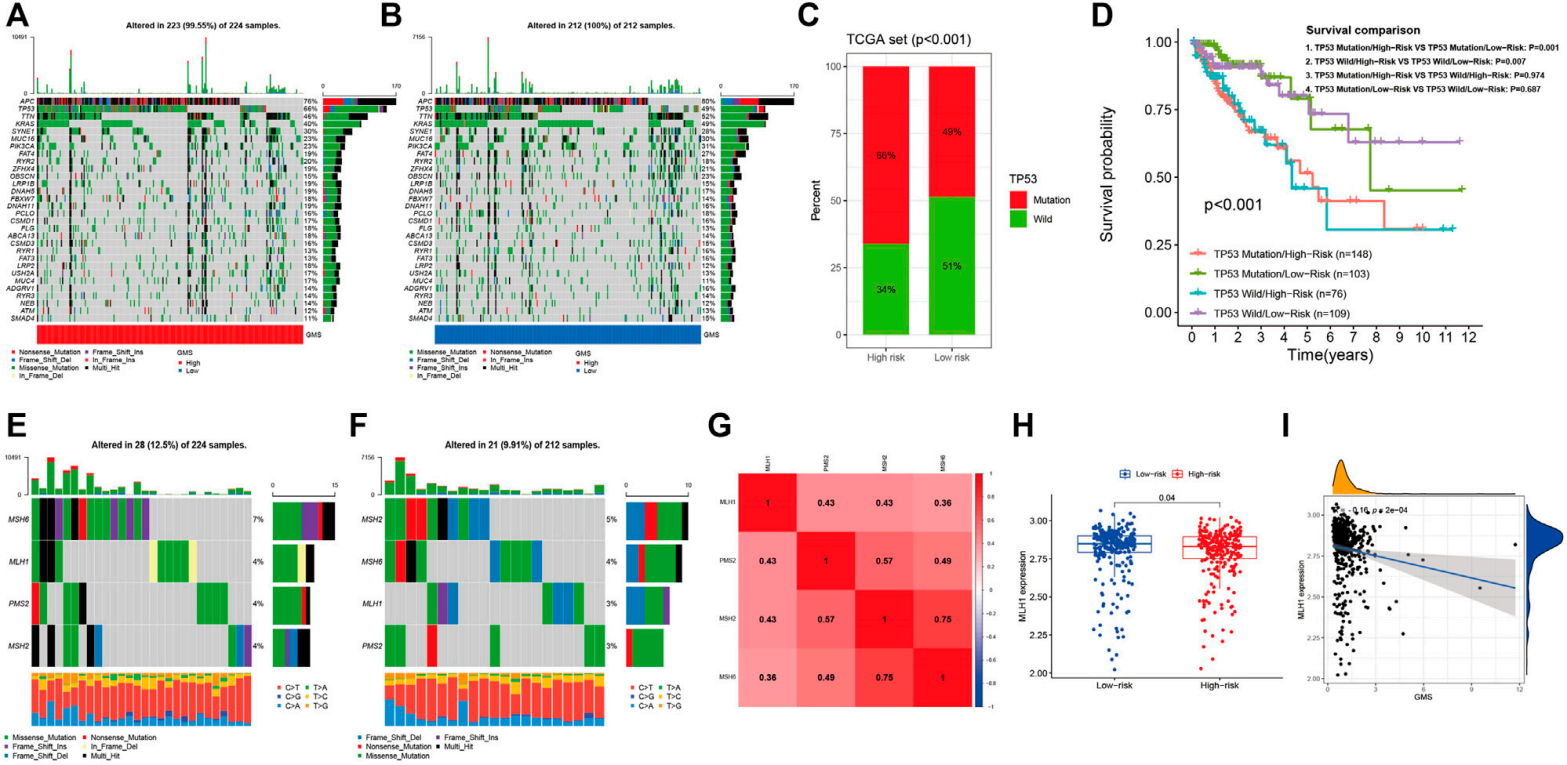
The landscape of somatic mutation and four characteristic genes of MMR system. **(A,B)**: The waterfall demonstrating the top 30 genes with highest mutation frequencies. **(C)**: The bar plot indicating that the mutational frequencies of TP53 in the high-risk group were higher than that in the low-risk group. **(D)**: The K-M survival curves stratified by the five-GMRHG prognostic signature and TP53 mutational status (TP53 wild and TP53 mutation). The overall survival of TCGA CRC patients with TP53 Mutation/High-risk was lower than that with TP53 mutation/Low-risk with statistical differences (*p* = 0.001). The overall survival of TCGA CRC patients with TP53 Wild/High-risk was lower than that with TP53 Wild/Low-risk with statistical differences (*p* = 0.007). Moreover, there were no statistical differences of the overall survival between the TCGA CRC patients in the high- or low-risk group with different mutation status (TP53 Mutation/High-risk VS TP53 Wild/High-risk: *p* = 0.974; TP53 Mutation/Low-risk VS TP53 Wild/Low-risk: *p* = 0.687). **(E,F)**: The waterfall demonstrating the mutational frequencies of MSH6, MLH1, PMS2, and MSH2 in the high- and low-risk high group. **(G)**: The co-expression correlations among MSH6, MLH1, PMS2, and MSH2. **(H)**: The expression difference of MLH1 in the high- and low-risk groups. **(I)**: The positive liner correlation between the expression of MLH1 and the GMS.

Figures 9A,B display the top 30 genes with highest mutation frequencies, among which TP53 showed the most significant statistical difference between the high- and low-risk groups (66 vs. 49%, *p* < 0.001) (Figure 9C). In order to further investigate the prognostic significance of the five-GMRHG prognostic signature in the TP53 mutational status, we conducted a hierarchical survival analysis for TP53-wild and TP53-mutation CRC patients, and found that the overall survival of TP53-mutation CRC patients in the high-risk group was lower than that in the low-risk group with a significant statistical difference (*p* = 0.001). Meanwhile, the overall survival of TP53-wild CRC patients exhibited the similar tendency to that of the overall survival of TP53-mutation CRC patients in the high- and low-risk groups (Figure 9D). Furthermore, we investigated the relationship between the MMR system and five-GMRHG prognostic signature. As shown in Figures 9E,F, MSH6 and MSH2 respectively demonstrated the highest mutation frequency in the high- and low-risk groups. Additionally, Pearson correlation analysis for the four characteristic genes of MMR system illustrated the underlying co-expression correlation among them, where MSH6-MSH2 displayed most fervent positive co-expression correlation (Figure 9G). The results of Wilcoxon ranking test for the expression differences of MSH6, MSH2, PMS2, and MLH1 indicated that the expression of MLH1 in the low-risk group was higher than that in the high-risk group (Figure 9H). The linear correlation conducted with Spearman correlation analysis also verified the negative relationship between the GMS and MLH1 expression (*R* = −0.16, *p* = 2e-04) (Figure 9I).

### Gene Set Enrichment Analysis

**FIGURE 10:**
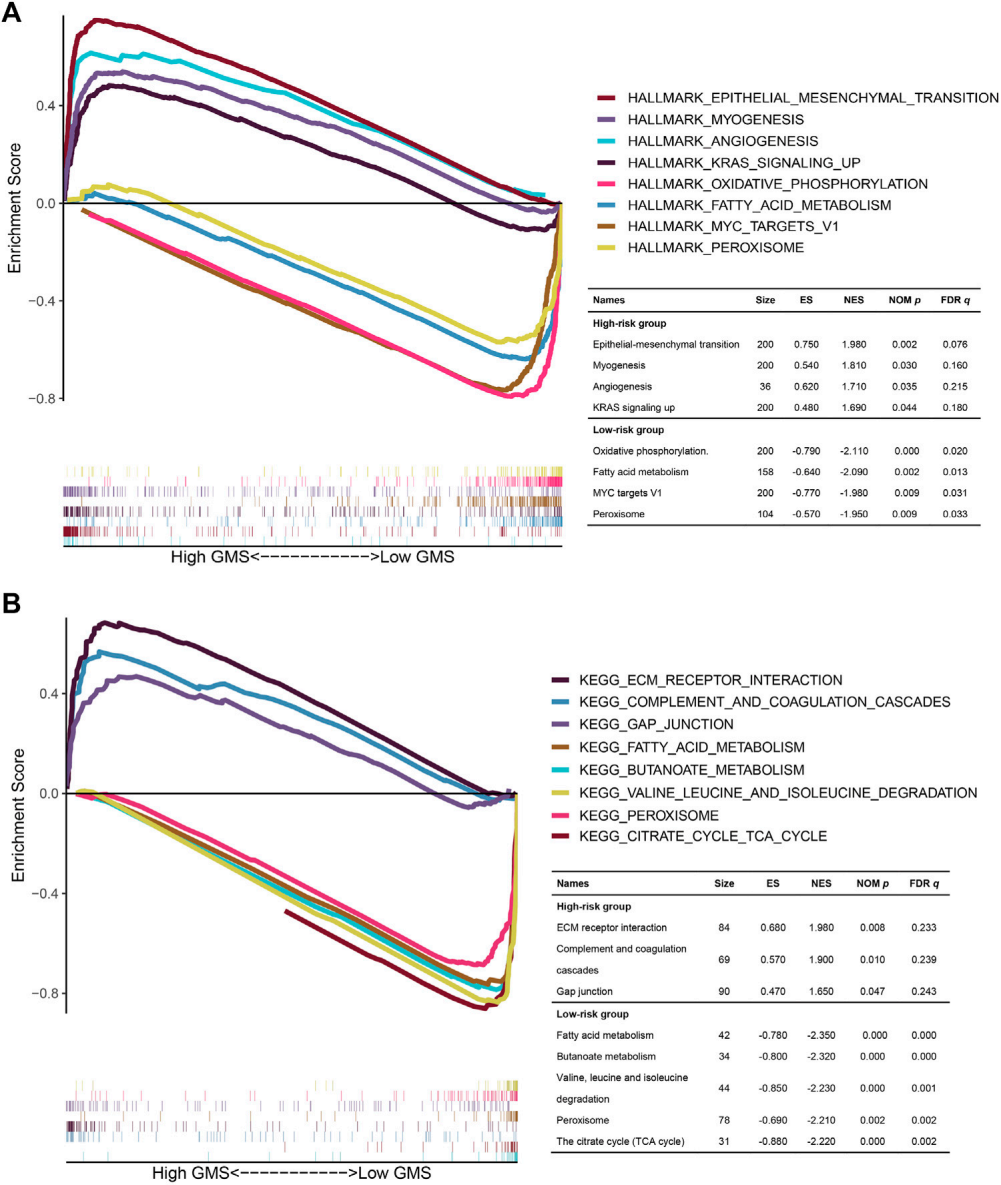
The GSEA for the TCGA CRC patients in the high- and low-risk groups. **(A,B)**: The multi-GSEA plots depicting the hallmark and KEGG functional pathways enriched in the gene matrices of TCGA CRC patients in the high- and low-risk groups. The curves above the X-axis represented the functional pathways enriched in the gene matrices of TCGA CRC patients in the high-risk group. Correspondingly, the curves below the X-axis represented the functional pathways enriched in the gene matrices of TCGA CRC patients in the low-risk group.

Targeting to gene expression matrix in the high- and low-risk groups, we performed GSEA to hierarchically reveal the underlying biological function in the TCGA CRC patients. In the results of the GSEA for the HALLMARK functional pathways (Figure 10A), the gene expression matrix in the high-risk group was mainly enriched in the frequently investigated cancer-related pathways, such as epithelial-mesenchymal transition (NOM *p* = 0.002, FDR *q* = 0.076), angiogenesis (NOM *p* = 0.035, FDR *q* = 0.215), and KRAS signaling up (NOM *p* = 0.044, FDR *q* = 0.180), while the gene expression matrix in the low-risk group was mainly enriched in the metabolism-related pathways, namely, oxidative phosphorylation (NOM *p* = 0.000, FDR *q* = 0.020) and fatty acid metabolism (NOM *p* = 0.002, FDR *q* = 0.013). In the results of the GSEA for the KEGG functional pathways (Figure 10B), the gene expression matrix in the high-risk group was mainly enriched in ECM receptor interaction (NOM *p* = 0.008, FDR *q* = 0.233), complement and coagulation cascades (NOM *p* = 0.010, FDR *q* = 0.239), and gap junction (NOM *p* = 0.047, FDR *q* = 0.243), while the gene expression matrix in the low-risk group was mainly enriched in 33 KEGG functional pathways, among which four of the top five pathways with the highest NES value adhering to the filtering standard (NOM *p* < 0.05, FDR *q* < 0.25) were mostly metabolism-related pathways (fatty acid metabolism (NOM *p* = 0.000, FDR *q* = 0.000), butanoate metabolism (NOM *p* = 0.000, FDR *q* = 0.000), valine leucine and isoleucine degradation (NOM *p* = 0.000, FDR *q* = 0.001), and citrate cycle (TCA cycle) (NOM *p* = 0.000, FDR *q* = 0.002). We also noted that in the analysis of HALLMARK and KEGG, the gene expression matrix in the low-risk group was both enriched in fatty acid metabolism and peroxisome, indicating that the CRC patients in the low-risk group might benefit from intervention against the fatty acid metabolism and peroxisome (HALLMARK: NOM *p* = 0.002, FDR *q* = 0.013; KEGG: NOM *p* = 0.002, FDR *q* = 0.002).

## Discussion

There are growing number of evidences indicated that an anomaly of lipid metabolism level contributes to the malignancy progression. As the result of the high proliferative activities, tumor cells are inclined to obtain the lipids with *de novo* synthesis, which gives rise to abnormal levels of lipase and signal transduction factors (Bao et al., 2016; Ru et al., 2017). Therefore, analyzing the differences in expression patterns of lipid metabolic mediators between normal samples and tumor samples would be conducive to determine the specific biological markers for the tumor diagnosis (Day et al., 2013).

In this study, 175 differential lipid metabolites were subjected to univariate and multivariate Cox regression analysis and we identified six lipid metabolites of the prognostic signature, including five high-risk lipid metabolites, namely, Cer(d18:0/14:0), LysoPE[22:6(4Z,7Z,10Z,13Z,16Z, 19Z)/0:0], PS [20:4(5Z,8Z,11Z, 14Z)/14:1(9Z)], PA[20:3(5Z,8Z, 11Z)/24:1(15Z)], and Ganglioside GT3[d18:0/18:1(9Z)], and one low-risk lipid metabolites (Substance P). Ceramide (Cer), one of the central active components of sphingolipid, is mainly composed of sphingoid long-chain base (LCB) and amide-linked to a fatty acyl chain (Ogretmen, 2018). Cer could be synthesized from serine and palmitate with *de novo* synthesis, and it could induce the apoptosis of cancer cells (Kramer et al., 2015) and protect cancer cells from apoptosis (Mesicek et al., 2010). Judith et al. found that the overexpression of CerS2 increased production of C(24:0) ceramide, which could protect cells from apoptosis induced by ionizing radiation (IR), and C(16:0) ceramide produced by the overexpression of CERS5 and CERS6, which contributed to IR-mediated apoptosis in HeLa cells(31). In this study, we found that Cer(d18:0/14:0) was a high-risk factor to predict the prognosis of CRC, also indirectly proving the role of Cer(d18:0/14:0) in the progression of CRC. LysoPE is the hemolytic metabolic product of phosphatidylethanolamine (Park et al., 2013). It has been shown that LysoPE was involved in intercellular signal transduction through G protein coupled receptor (GPCR) (Park et al., 2013). The level of LysoPE in colon cancer tissue is significantly higher than that in normal colon epithelium (Hofmanová et al., 2021). This study further found that the level of LysoPE in mucinous adenocarcinoma was higher than that in adenocarcinoma at the serum level, which indirectly confirmed that LysoPE, as a high-risk factor, could predict the prognosis of CRC patients. Phosphatidylserine (PS), the only kind of phospholipid that could regulate the functional state of critical proteins in cell membrane, distributed on the vesicle membrane and participated in the process of related diseases (Skotland et al., 2019; Wodlej et al., 2019). Ran et al. emphasized that the exposure of PS in tumor cells was induced by oxidative stress and cytokines, and the exposed PS binding to immune cells regulated the immune infiltrations and biological process of tumor cells (Ran and Thorpe, 2002). Previous studies have shown that fatty acid chain elongation, one of the characteristics of malignant tumors, was represented by PS in prostate cancer (Butler et al., 2021). In CRC, Jiřina et al. utilized LC-MS to determine phospholipid components, including phosphatidylserine as the biological markers to distinguish normal colorectal epithelial cells from CRC cells. The clinical correlation analysis also suggested that the level of PS was higher in the advanced pathological stage (stage Ⅲ-Ⅳ) and the mucinous adenocarcinoma, which further demonstrated that PS was a high-risk factor affecting the prognosis of patients with colorectal cancer. Phosphatidic acid (PA) was found to be a high-risk factor in this study. Although it is a type of membrane phospholipid with a simple structure and it accounted for a minority of membrane lipid components, it exists in the cell membrane of almost all cells (Young et al., 2010; Kulig et al., 2019). Previous studies have shown that PA is mostly correlated with apoptosis regulatory signals that promote the proliferation of tumor cells. Zhao et al. proposed that the combination of PA and tBid would enhance lysosomal membrane permeabilization (LMP), thus triggering the apoptotic signaling (Zhao et al., 2012). Moreover, Taga et al. found that PA stimulated the mTORC1 signaling pathway to protect human neuroblastoma cells against apoptosis induced by oxygen stress (Taga et al., 2011). The latest study further confirmed that PA has been proven to be one of the key lipid signals mediating the activation of classic cancer signaling pathway-Hippo pathway (Han et al., 2018). Ganglioside GT3 is a kind of ganglioside mainly composed of glycosphingolipids and sialic acids linking to sugar chain (Labrada et al., 2018). It is a type of component concentrating on the lipid raft in the plasma membrane (Campos et al., 2021). The main difference among more than 60 known gangliosides depends on the locations and numbers of NANA residues (Fuse et al., 1982). Previous studies have shown that ganglioside GD2/3 (Yoshida et al., 2001) and GM3 (Hayashi et al., 2013) played important roles in tumor cell invasions and metastasis, but the role of ganglioside GT3 in malignancies including CRC has not been clearly elaborated. This study found that ganglioside GT3 was a high-risk factor for predicting the prognosis of CRC, and it might also provide inspiration for the role of ganglioside GT3 in CRC. SP is a member of tachykinin peptide family. It is located in the central nervous system and other several peripheral tissues, including the entire length of the gastrointestinal and colorectal tract. The biological effect of SP is mediated by three different G protein coupled receptors (GPCR), which were neurokinin (NK)-1, 2 and 3, respectively. SP had high affinity for NK-1 receptor (NK-1R) and low affinity for NK-2 and 3 receptors (Muñoz and Coveñas, 2020). Reubi et al. found that the levels of SP and NK1R in CRC tissue were higher than those in normal tissue (Lorestani et al., 2020). The SP content in proximal vein of CRC tissue was three to five times higher than that in distal vein of CRC tissue, suggesting that the distribution and regulation mechanism of SP in vascular bed are closely related to the mechanism of CRC metastasis (Reubi et al., 1996). The clinical correlation analysis of this study revealed that the level of SP in adenocarcinoma was higher than that in mucinous adenocarcinoma. SP as a low-risk factor to predict the prognosis of CRC patients might be related with this. Moreover, we utilized the independent prognostic factors to plot a nomogram to systematically evaluate the overall survival of CRC patients. The calibration curve and ROC curve indicated that the nomogram had favorable clinical application and accuracy in predicting the prognosis of CRC patients. Furthermore, through functional enrichment analysis, we found that 175 kinds of differential lipid metabolites were significantly enriched in glycerophospholipid metabolism with statistical significance (FDR = 0.0016, Impact = 0.403). Glycerophospholipids, the most abundant membrane lipids, are composed of glycerol backbone, two fatty acid chains, and a polar head group, which mainly produces phospholipid substances in human cells, such as phosphatidylserine (PS), phosphatidylcholine (PC), phosphatidylethanolamine (PE), phosphatidylglycerol (PG), and phosphatidylinositol (PI). However, for now, there is no systematic exposition on the guiding role of glycerophospholipid metabolism in the prognosis of CRC patients. Herein, we have elaborately expounded the prognostic and molecular biological functions of glycerophospholipid metabolism from the transcriptomic level. Integrated with the prognostic information of 877 CRC patients, we developed the five-GMRHG (ACOX1, ATOH1, CPT2, PCSK5, and TINCR) prognostic signature to conduct stratified prognostic analysis for CRC patients. Of the five GMRHGs, PCSK5 and TINCR were high-risk GMRHGs and ACOX1, ATOH1, and CPT2 were low-risk GMRHGs. ACOX1, as a key enzyme involved in fatty acid oxidation (FAO) pathway, has been proved to be downregulated by p38MAPK/PPARα signaling pathway regulated by CD147 (a transmembrane protein regulated by lipid metabolism), thus inhibiting fatty acid-β oxidation (Li et al., 2015). Sun et al. have also found that ACOX1 overexpression weakened the enhancement of miR-15b-5p overexpression on the migration and invasion of CRC cells, and SIRT1/miR-15b5p/ACOX1 axis played an important role in the metastasis of CRC (Sun et al., 2017). ATOH1 is a member of the basic helix-loop-helix (BHLH) family of transcription factors (Zheng et al., 2011). Previous studies have shown that ATOH1 played an important role in the differentiation of intestinal secretory cells. Shroyer et al. found the loss of intestinal Paneth, goblet cells, and intestinal endocrine cells in adult rats after knocking down ATOH1 (Shroyer et al., 2007). Recent studies have shown that the overexpression of ATOH1 promoted functional intestinal stem cells to differentiate into secretory cells, and its expression was regulated by LKB1 (Gao et al., 2020). In the clinical correlation analysis, CPT2 exhibited higher expression levels in the early pathological stage and N staging, which could support the role of CPT2 as a low-risk GMRHG for CRC. PCSK5 is a type of proprotein convertase that can affect the metastatic property of CRC by participating in the production of adhesion molecules and growth factors (Bontemps et al., 2007). It has been proven that abnormally upregulated expression of PCSK5 indicates increased metastatic potential of CRC (Scamuffa et al., 2008). TINCR is a key lncRNA involved in the stability of mRNA which is responsible for the maintenance of epidermal tissue differentiation (Kretz et al., 2013). Yu et al. reported that TINCR expression level could be independently prognostic for CRC, and its abnormally upregulated expression was associated with more adverse outcomes. In the meantime, they also found that *in vitro* knockout of TINCR contributed to significantly decreased levels of cancer cell proliferation, while the overexpression of TINCR was conducive to cancer progression with the involvement of miR-7-5p and PI3K/Akt/mTOR signaling (Yu et al., 2019). In the present study, CRC patients were grouped by the median GMS was proven to be significant in prognostic prediction with K-M survival analysis, independent prognostic analysis, and time-dependent ROC curves. Additionally, GSEA pathway analysis was performed and revealed much more highly activated pathways involved in epithelial-mesenchymal transition (EMT), angiogenesis, and KRAS signaling up in the high-risk group. It is known that EMT is active in the presence of most tumors, especially epithelial tumors. It is characterized by the gradual loss of epithelial properties while gaining mesenchymal properties as the tumor progresses (Grünert et al., 2003; Dongre and Weinberg, 2019), and it has been proven to have a close relationship with the distant metastasis of CRC (Lambert et al., 2017). Early in the 1970s, Folkman et al. reported that the occurrence and progression of tumors were accompanied by active angiogenesis (Folkman, 1971). Vascular endothelial growth factor (VEGF) is a typical gene involved in angiogenesis. Ferrara et al. revealed that suppression of VEGF expression could decrease vascular density and block tumor progression (Kim et al., 1993). Application of first-line anti-vascular agent Bevacizumab was conducive to largely prolonging the progression-free survival (PFS) of patients with metastatic CRC (Cunningham et al., 2013). Aashiq et al. found that PI3K signaling was crucial in tumor progression by mediating lipid metabolism and glycolysis. They reported that blockade of the PI3K signaling suppressed CRC cell migration and invasion following reductions in metabolic flux and tumor angiogenesis (Hussain et al., 2016). EMT and angiogenesis are tightly correlated in oncogenesis and metastasis (Sánchez-García, 2009), while the crosstalk between VEGF and Notch, an epidermal cell proliferation suppressor, under hypoxic stimuli can augment the EMT in cancer cells (Holderfield and Hughes, 2008). Here, EMT and angiogenesis were active in the high-risk group, indicative that the two biological functions might be much more significant in tumor progression of the high-risk patients.

Further, the characteristics depicting TME and somatic mutation map were hierarchically viewed for the CRC patients. It was revealed that aDCs, CD8+_T_cells, macrophages, T_helper_cells, Tfh, Th1_cells, TIL, CCR, check-point, HLA, inflammation-promoting, T_cell_co-inhibition, and T_cell_co-stimulation exhibited higher levels in the high-risk group versus the low-risk group with statistical differences. Meanwhile, it indirectly demonstrated that CRC patients in the high-risk had higher immune activities. Moreover, in the previous research on melanoma, it was found that the immune escape was realized based on T cells in the TME. Specifically, T cells target to recognize tumor antigen followed by upregulating PD-1 and expressing IFN-γ which can contribute to expressing PD-L1 by immune and tumor cells. In that setting, the expressed PD-L1 will bind to PD-1 to block T cell surveillance, ultimately leading to immune escape (Taube et al., 2012). Here, the high-risk group had an increase in the expression of IFN-γ, PD-1, and PD-L1 as analyzed by the TIDE algorithm, and it was more likely to experience immune escape. Similar processes could also occur in CRC. Somatic mutational analysis showed that mutations in APC, TP53, TTN, KRAS, and SYNE were highly frequent in both high- and low-risk groups. In most cases, biallelic inactivation of APC is the culprit for CRC occurrence and it plays a key role in tumor progression to adenomas by activating responsible pathways (Fennell et al., 2020). It is reported that CRC progression is largely associated with the mutations in KRAS and TP53 genes (Eklöf et al., 2013; Fennell et al., 2020). KRAS encodes K-Ras protein, which belongs to the Ras-Raf-MEK-ERK signaling pathway, thought to be responsible for CRC growth and proliferation (Eklöf et al., 2013). TP53 is a tumor suppressor that is abnormally mutated to CRC progression. Additionally, TP53 mutations can be independently prognostic for the outcome of CRC, predicting more adverse outcomes as well as more significant resistance to chemotherapeutics (Li et al., 2019). We here noted that the mutation frequency of TP53 in high-risk group was much higher versus that of the low-risk group, which was statistically significant. In the K-M survival analysis, CRC patients in the high-risk had a shorter survival under the same TP53 mutation frequency. This suggests that the GMS-based grouping method is superior to the conventional TP53-directed method for predicting the overall survival of CRC patients. MMR includes MLH1, MSH2, MSH6, and PMS2. Mutations or modifications (such as methylation) of their upstream genes responsible for protein synthesis can lead to defects of the MMR genes. In that circumstance, the replication errors in MS areas fail to get corrected, leading to error accumulation and the subsequent occurrence of MSI (Sahin et al., 2019). It has been reported that MSI affects approximately 15–20% of CRC patients and is of vital significance in CRC initiation and development (Richman, 2015; Sahin et al., 2019). In this study, the MMR gene mutation frequency was 19% in the high-risk group, compared to 15% in the low-risk group, which is consistent with the previous finding. Among the four MMR genes, MLH1 was proven to be abnormally silenced in CRC, and the silencing or mutation of MLH1 might be crucial in tumor progression (Ma et al., 2016; Han et al., 2020). Moreover, MLH1 downregulation is associated with poor outcome of CRC and can be independently prognostic for PFS (Han et al., 2020). Further differential expression and Spearman correlational analysis revealed that MLH1 exhibited higher expression in the low-risk group relative to the high-risk group. This supported the low expression of MLH1 in the high-risk group and its prognostic significance for poor outcome of CRC.

In summary, we systematically investigated the role of lipid metabolism in monitoring the prognosis of CRC patients with metabonomics and transcriptomic methods, and constructed a composite nomogram individually predicting the overall survival of CRC patients based on the independent prognostic factors (six-lipid-metabolite prognostic signature, pathological stage, histological type, and T staging). The five-GMRHG prognostic signature can not only be used to predict the prognosis of CRC patients, but also shed light on the CRC patients’ current immune infiltration status, somatic mutational landscape, and potential biological functions, and provided inspiration for individualized treatment of CRC patients in the future.

## Data Availability

The datasets presented in this study can be found in the MetaboLights repository, accession number MTBLS3945.

## References

[B1] Angelova M. Charoentong P. Hackl H. Fischer M. L. Snajder R. Krogsdam A. M. (2015). Characterization of the Immunophenotypes and Antigenomes of Colorectal Cancers Reveals Distinct Tumor Escape Mechanisms and Novel Targets for Immunotherapy. Genome Biol. 16, 64. 10.1186/s13059-015-0620-6 25853550 PMC4377852

[B2] Aran V. Victorino A. P. Thuler L. C. Ferreira C. G. (2016). Colorectal Cancer: Epidemiology, Disease Mechanisms and Interventions to Reduce Onset and Mortality. Clin. Colorectal Cancer 15, 195–203. 10.1016/j.clcc.2016.02.008 26964802

[B3] Bao J. Zhu L. Zhu Q. Su J. Liu M. Huang W. (2016). SREBP-1 Is an Independent Prognostic Marker and Promotes Invasion and Migration in Breast Cancer. Oncol. Lett. 12, 2409–2416. 10.3892/ol.2016.4988 27703522 PMC5038874

[B4] Barbie D. A. Tamayo P. Boehm J. S. Kim S. Y. Moody S. E. Dunn I. F. (2009). Systematic RNA Interference Reveals that Oncogenic KRAS-Driven Cancers Require TBK1. Nature 462, 108–112. 10.1038/nature08460 19847166 PMC2783335

[B5] Bontemps Y. Scamuffa N. Calvo F. Khatib A.-M. (2007). Potential Opportunity in the Development of New Therapeutic Agents Based on Endogenous and Exogenous Inhibitors of the Proprotein Convertases. Med. Res. Rev. 27, 631–648. 10.1002/med.20072 17019676 PMC7168524

[B6] Butler L. M. Mah C. Y. Machiels J. Vincent A. D. Irani S. Mutuku S. M. (2021). Lipidomic Profiling of Clinical Prostate Cancer Reveals Targetable Alterations in Membrane Lipid Composition. Cancer Res. 81, 4981–4993. 10.1158/0008-5472.CAN-20-3863 34362796

[B7] Campos F. S. O. Piña-Rodrigues F. M. Reis A. Atella G. C. Mermelstein C. S. Allodi S. (2021). Lipid Rafts from Olfactory Ensheathing Cells: Molecular Composition and Possible Roles. Cell Mol Neurobiol 41, 525–536. 10.1007/s10571-020-00869-4 32415577 PMC11448638

[B8] Chong J. Soufan O. Li C. Caraus I. Li S. Bourque G. (2018). MetaboAnalyst 4.0: towards More Transparent and Integrative Metabolomics Analysis. Nucleic Acids Res. 46, W486–W494. 10.1093/nar/gky310 29762782 PMC6030889

[B9] Cunningham D. Lang I. Marcuello E. Lorusso V. Ocvirk J. Shin D. B. (2013). Bevacizumab Plus Capecitabine versus Capecitabine Alone in Elderly Patients with Previously Untreated Metastatic Colorectal Cancer (AVEX): an Open-Label, Randomised Phase 3 Trial. Lancet Oncol. 14, 1077–1085. 10.1016/S1470-2045(13)70154-2 24028813

[B10] Day S. D. Enos R. T. McClellan J. L. Steiner J. L. Velázquez K. T. Murphy E. A. (2013). Linking Inflammation to Tumorigenesis in a Mouse Model of High-Fat-Diet-Enhanced colon Cancer. Cytokine 64, 454–462. 10.1016/j.cyto.2013.04.031 23735174 PMC4826024

[B11] De Schrijver E. Brusselmans K. Heyns W. Verhoeven G. Swinnen J. V. (2003). RNA Interference-Mediated Silencing of the Fatty Acid Synthase Gene Attenuates Growth and Induces Morphological Changes and Apoptosis of LNCaP Prostate Cancer Cells. Cancer Res. 63, 3799–3804. 12839976

[B12] DeLaney K. Sauer C. Vu N. Li L. (2018). Recent Advances and New Perspectives in Capillary Electrophoresis-Mass Spectrometry for Single Cell "Omics". Molecules 24, 42. 10.3390/molecules24010042 PMC6337428 30583525

[B13] Dongre A. Weinberg R. A. (2019). New Insights into the Mechanisms of Epithelial-Mesenchymal Transition and Implications for Cancer. Nat. Rev. Mol. Cel Biol 20, 69–84. 10.1038/s41580-018-0080-4 30459476

[B14] Eklöf V. Wikberg M. L. Edin S. Dahlin A. M. Jonsson B.-A. Öberg Å. (2013). The Prognostic Role of KRAS, BRAF, PIK3CA and PTEN in Colorectal Cancer. Br. J. Cancer 108, 2153–2163. 10.1038/bjc.2013.212 23660947 PMC3670497

[B15] Fennell L. J. Kane A. Liu C. McKeone D. Fernando W. Su C. (2020). APC Mutation Marks an Aggressive Subtype of BRAF Mutant Colorectal Cancers. Cancers 12, 1171. 10.3390/cancers12051171 PMC7281581 32384699

[B16] Folkman J. (1971). Tumor Angiogenesis: Therapeutic Implications. N. Engl. J. Med. 285, 1182–1186. 10.1056/NEJM197111182852108 4938153

[B17] Fuse A. Handa S. Kuwata T. (1982). Effect of Glycolipids and Glycophorin on the Activity of Human Interferon-β and -γ. Antiviral Res. 2, 161–166. 10.1016/0166-3542(82)90018-3 6814359

[B18] Gao P. Zhou C. Zhao L. Zhang G. Zhang Y. (2016). Tissue Amino Acid Profile Could Be Used to Differentiate Advanced Adenoma from Colorectal Cancer. J. Pharm. Biomed. Anal. 118, 349–355. 10.1016/j.jpba.2015.11.007 26595283

[B19] Gao Y. Yan Y. Tripathi S. Pentinmikko N. Amaral A. Päivinen P. (2020). LKB1 Represses ATOH1 via PDK4 and Energy Metabolism and Regulates Intestinal Stem Cell Fate. Gastroenterology 158, 1389–1401. 10.1053/j.gastro.2019.12.033 31930988

[B20] Grünert S. Jechlinger M. Beug H. (2003). Diverse Cellular and Molecular Mechanisms Contribute to Epithelial Plasticity and Metastasis. Nat. Rev. Mol. Cel Biol 4, 657–665. 10.1038/nrm1175 12923528

[B21] Han H. Qi R. Zhou J. J. Ta A. P. Yang B. Nakaoka H. J. (2018). Regulation of the Hippo Pathway by Phosphatidic Acid-Mediated Lipid-Protein Interaction. Mol. Cel 72, 328–340. 10.1016/j.molcel.2018.08.038 PMC6195446 30293781

[B22] Han Y. Peng Y. Fu Y. Cai C. Guo C. Liu S. (2020). MLH1 Deficiency Induces Cetuximab Resistance in Colon Cancer via Her‐2/PI3K/AKT Signaling. Adv. Sci. 7, 2000112. 10.1002/advs.202000112 PMC7341094 32670759

[B23] Hänzelmann S. Castelo R. Guinney J. (2013). GSVA: Gene Set Variation Analysis for Microarray and RNA-Seq Data. BMC Bioinformatics 14, 7. 10.1186/1471-2105-14-7 23323831 PMC3618321

[B24] Hayashi N. Chiba H. Kuronuma K. Go S. Hasegawa Y. Takahashi M. (2013). Detection of N-Glycolyated Gangliosides in Non-small-cell Lung Cancer Using GMR8 Monoclonal Antibody. Cancer Sci. 104, 43–47. 10.1111/cas.12027 23004020 PMC7657197

[B25] Hofmanová J. Slavík J. Ciganek M. Ovesná P. Tylichová Z. Karasová M. (2021). Complex Alterations of Fatty Acid Metabolism and Phospholipidome Uncovered in Isolated Colon Cancer Epithelial Cells. Ijms 22, 6650. 10.3390/ijms22136650 34206240 PMC8268957

[B26] Holderfield M. T. Hughes C. C. W. (2008). Crosstalk between Vascular Endothelial Growth Factor, Notch, and Transforming Growth Factor-β in Vascular Morphogenesis. Circ. Res. 102, 637–652. 10.1161/CIRCRESAHA.107.167171 18369162

[B27] Hung Y.-H. Chan Y.-S. Chang Y.-S. Lee K.-T. Hsu H.-P. Yen M.-C. (2014). Fatty Acid Metabolic Enzyme Acyl-CoA Thioesterase 8 Promotes the Development of Hepatocellular Carcinoma. Oncol. Rep. 31, 2797–2803. 10.3892/or.2014.3155 24788990

[B28] Hussain A. Qazi A. K. Mupparapu N. Guru S. K. Kumar A. Sharma P. R. (2016). Modulation of Glycolysis and Lipogenesis by Novel PI3K Selective Molecule Represses Tumor Angiogenesis and Decreases Colorectal Cancer Growth. Cancer Lett. 374, 250–260. 10.1016/j.canlet.2016.02.030 26921131

[B29] Jiang P. Gu S. Pan D. Fu J. Sahu A. Hu X. (2018). Signatures of T Cell Dysfunction and Exclusion Predict Cancer Immunotherapy Response. Nat. Med. 24, 1550–1558. 10.1038/s41591-018-0136-1 30127393 PMC6487502

[B30] Kim K. J. Li B. Winer J. Armanini M. Gillett N. Phillips H. S. (1993). Inhibition of Vascular Endothelial Growth Factor-Induced Angiogenesis Suppresses Tumour Growth *In Vivo* . Nature 362, 841–844. 10.1038/362841a0 7683111

[B31] Kitahara C. M. Berrington de González A. Freedman N. D. Huxley R. Mok Y. Jee S. H. (2011). Total Cholesterol and Cancer Risk in a Large Prospective Study in Korea. Jco 29, 1592–1598. 10.1200/JCO.2010.31.5200 PMC3082977 21422422

[B32] Kramer R. Bielawski J. Kistner‐Griffin E. Othman A. Alecu I. Ernst D. (2015). Neurotoxic 1‐deoxysphingolipids and Paclitaxel‐induced Peripheral Neuropathy. FASEB j. 29, 4461–4472. 10.1096/fj.15-272567 26198449 PMC4608911

[B33] Kretz M. Siprashvili Z. Chu C. Webster D. E. Zehnder A. Qu K. (2013). Control of Somatic Tissue Differentiation by the Long Non-coding RNA TINCR. Nature 493, 231–235. 10.1038/nature11661 23201690 PMC3674581

[B34] Kulig W. Korolainen H. Zatorska M. Kwolek U. Wydro P. Kepczynski M. (2019). Complex Behavior of Phosphatidylcholine-Phosphatidic Acid Bilayers and Monolayers: Effect of Acyl Chain Unsaturation. Langmuir 35, 5944–5956. 10.1021/acs.langmuir.9b00381 30942590

[B35] Labrada M. Dorvignit D. Hevia G. Rodríguez-Zhurbenko N. Hernández A. M. Vázquez A. M. (2018). GM3(Neu5Gc) Ganglioside: an Evolution Fixed Neoantigen for Cancer Immunotherapy. Semin. Oncol. 45, 41–51. 10.1053/j.seminoncol.2018.04.003 30318083

[B36] Lambert A. W. Pattabiraman D. R. Weinberg R. A. (2017). Emerging Biological Principles of Metastasis. Cell 168, 670–691. 10.1016/j.cell.2016.11.037 28187288 PMC5308465

[B37] Langfelder P. Horvath S. (2008). WGCNA: an R Package for Weighted Correlation Network Analysis. BMC Bioinformatics 9, 559. 10.1186/1471-2105-9-559 19114008 PMC2631488

[B38] Li H. Zhang J. Tong J. H. M. Chan A. W. H. Yu J. Kang W. (2019). Targeting the Oncogenic P53 Mutants in Colorectal Cancer and Other Solid Tumors. Ijms 20, 5999. 10.3390/ijms20235999 PMC6929124 31795192

[B39] Li J. Huang Q. Long X. Zhang J. Huang X. Aa J. (2015). CD147 Reprograms Fatty Acid Metabolism in Hepatocellular Carcinoma Cells through Akt/mTOR/SREBP1c and P38/PPARα Pathways. J. Hepatol. 63, 1378–1389. 10.1016/j.jhep.2015.07.039 26282231

[B40] Liao F. He W.-z. Jiang C. Yin C.-x. Guo G.-f. Chen X. (2015). A High LDL-C to HDL-C Ratio Predicts Poor Prognosis for Initially Metastatic Colorectal Cancer Patients with Elevations in LDL-C. Ott 8, 3135–3142. 10.2147/OTT.S90479 PMC4629979 26604782

[B41] Lodi A. Tiziani S. Khanim F. L. Günther U. L. Viant M. R. Morgan G. J. (2013). Proton NMR-Based Metabolite Analyses of Archived Serial Paired Serum and Urine Samples from Myeloma Patients at Different Stages of Disease Activity Identifies Acetylcarnitine as a Novel Marker of Active Disease. PLoS One 8, e56422. 10.1371/journal.pone.0056422 23431376 PMC3576408

[B42] Lorestani S. Ghahremanloo A. Jangjoo A. Abedi M. Hashemy S. I. (2020). Evaluation of Serum Level of Substance P and Tissue Distribution of NK-1 Receptor in Colorectal Cancer. Mol. Biol. Rep. 47, 3469–3474. 10.1007/s11033-020-05432-4 32277443

[B43] Lupu R. Menendez J. (2006). Pharmacological Inhibitors of Fatty Acid Synthase (FASN)-Catalyzed Endogenous Fatty Acid Biogenesis: A New Family of Anti-cancer Agents? Cpb 7, 483–494. 10.2174/138920106779116928 17168665

[B44] Ma G. Ge Y. Gu D. Du M. Chu H. Chen J. (2016). Functional Annotation of Colorectal Cancer Susceptibility Loci identifiesMLH1rs1800734 Associated with MSI Patients. Gut 65, 1227–1228. 10.1136/gutjnl-2016-311543 26911399

[B45] Mesicek J. Lee H. Feldman T. Jiang X. Skobeleva A. Berdyshev E. V. (2010). Ceramide Synthases 2, 5, and 6 Confer Distinct Roles in Radiation-Induced Apoptosis in HeLa Cells. Cell Signal. 22, 1300–1307. 10.1016/j.cellsig.2010.04.006 20406683 PMC4348005

[B46] Muka T. Kraja B. Ruiter R. de Keyser C. E. Hofman A. Stricker B. H. (2016). Dietary Polyunsaturated Fatty Acids Intake Modifies the Positive Association between Serum Total Cholesterol and Colorectal Cancer Risk: the Rotterdam Study. J. Epidemiol. Community Health 70, 881–887. 10.1136/jech-2015-206556 26917548

[B47] Muñoz M. Coveñas R. (2020). Neurokinin Receptor Antagonism: a Patent Review (2014-present). Expert Opin. Ther. Patents 30, 527–539. 10.1080/13543776.2020.1769599 32401556

[B48] Ogretmen B. (2018). Sphingolipid Metabolism in Cancer Signalling and Therapy. Nat. Rev. Cancer 18, 33–50. 10.1038/nrc.2017.96 29147025 PMC5818153

[B49] Park S.-J. Lee K.-P. Kang S. Chung H.-Y. Bae Y.-S. Okajima F. (2013). Lysophosphatidylethanolamine Utilizes LPA1 and CD97 in MDA-MB-231 Breast Cancer Cells. Cell Signal. 25, 2147–2154. 10.1016/j.cellsig.2013.07.001 23838008

[B50] Ramakrishna M. Williams L. H. Boyle S. E. Bearfoot J. L. Sridhar A. Speed T. P. (2010). Identification of Candidate Growth Promoting Genes in Ovarian Cancer through Integrated Copy Number and Expression Analysis. PLoS One 5, e9983. 10.1371/journal.pone.0009983 20386695 PMC2851616

[B51] Ran S. Thorpe P. E. (2002). Phosphatidylserine Is a Marker of Tumor Vasculature and a Potential Target for Cancer Imaging and Therapy. Int. J. Radiat. Oncology*Biology*Physics 54, 1479–1484. 10.1016/s0360-3016(02)03928-7 12459374

[B52] Reubi J. Mazzucchelli L. Hennig I. Laissue J. (1996). Local Up-Regulation of Neuropeptide Receptors in Host Blood Vessels Around Human Colorectal Cancers. Gastroenterology 110, 1719–1726. 10.1053/gast.1996.v110.pm8964396 8964396

[B53] Richman S. (2015). Deficient Mismatch Repair: Read All about it (Review). Int. J. Oncol. 47, 1189–1202. 10.3892/ijo.2015.3119 26315971 PMC4583524

[B54] Rodriguez‐Broadbent H. Law P. J. Sud A. Palin K. Tuupanen S. Gylfe A. (2017). Mendelian Randomisation Implicates Hyperlipidaemia as a Risk Factor for Colorectal Cancer. Int. J. Cancer 140, 2701–2708. 10.1002/ijc.30709 28340513 PMC6135234

[B55] Ru P. Hu P. Geng F. Mo X. Cheng C. Yoo J. Y. (2017). Feedback Loop Regulation of SCAP/SREBP-1 by miR-29 Modulates EGFR Signaling-Driven Glioblastoma Growth. Cel Rep. 18, 1076–1077. 10.1016/j.celrep.2017.01.016 28122233

[B56] Sahin I. H. Akce M. Alese O. Shaib W. Lesinski G. B. El-Rayes B. (2019). Immune Checkpoint Inhibitors for the Treatment of MSI-H/MMR-D Colorectal Cancer and a Perspective on Resistance Mechanisms. Br. J. Cancer 121, 809–818. 10.1038/s41416-019-0599-y 31607751 PMC6889302

[B57] Sánchez-García I. (2009). The Crossroads of Oncogenesis and Metastasis. N. Engl. J. Med. 360, 297–299. 10.1056/NEJMcibr0808031 19144947

[B58] Santos C. R. Schulze A. (2012). Lipid Metabolism in Cancer. FEBS J. 279, 2610–2623. 10.1111/j.1742-4658.2012.08644.x 22621751

[B59] Scamuffa N. Siegfried G. Bontemps Y. Ma L. Basak A. Cherel G. (2008). Selective Inhibition of Proprotein Convertases Represses the Metastatic Potential of Human Colorectal Tumor Cells. J. Clin. Invest. 118, 352–363. 10.1172/jci32040 18064302 PMC2117762

[B60] Shroyer N. F. Helmrath M. A. Wang V. Y. C. Antalffy B. Henning S. J. Zoghbi H. Y. (2007). Intestine-specific Ablation of Mouse Atonal Homolog 1 (Math1) Reveals a Role in Cellular Homeostasis. Gastroenterology 132, 2478–2488. 10.1053/j.gastro.2007.03.047 17570220

[B61] Skotland T. Hessvik N. P. Sandvig K. Llorente A. (2019). Exosomal Lipid Composition and the Role of Ether Lipids and Phosphoinositides in Exosome Biology. J. Lipid Res. 60, 9–18. 10.1194/jlr.R084343 30076207 PMC6314266

[B62] Subramanian A. Tamayo P. Mootha V. K. Mukherjee S. Ebert B. L. Gillette M. A. (2005). Gene Set Enrichment Analysis: a Knowledge-Based Approach for Interpreting Genome-wide Expression Profiles. Proc. Natl. Acad. Sci. 102, 15545–15550. 10.1073/pnas.0506580102 16199517 PMC1239896

[B63] Sun L.-N. Zhi Z. Chen L.-Y. Zhou Q. Li X.-M. Gan W.-J. (2017). SIRT1 Suppresses Colorectal Cancer Metastasis by Transcriptional Repression of miR-15b-5p. Cancer Lett. 409, 104–115. 10.1016/j.canlet.2017.09.001 28923398

[B64] Taga M. Mouton-Liger F. Paquet C. Hugon J. (2011). Modulation of Oxidative Stress and Tau Phosphorylation by the mTOR Activator Phosphatidic Acid in SH-Sy5y Cells. FEBS Lett. 585, 1801–1806. 10.1016/j.febslet.2011.04.022 21510936

[B65] Taube J. M. Anders R. A. Young G. D. Xu H. Sharma R. McMiller T. L. (2012). Colocalization of Inflammatory Response with B7-H1 Expression in Human Melanocytic Lesions Supports an Adaptive Resistance Mechanism of Immune Escape. Sci. Transl. Med. 4, 127ra37. 10.1126/scitranslmed.3003689 PMC3568523 22461641

[B66] Tenori L. Oakman C. Morris P. G. Gralka E. Turner N. Cappadona S. (2015). Serum Metabolomic Profiles Evaluated after Surgery May Identify Patients with Oestrogen Receptor Negative Early Breast Cancer at Increased Risk of Disease Recurrence. Results from a Retrospective Study. Mol. Oncol. 9, 128–139. 10.1016/j.molonc.2014.07.012 25151299 PMC5528693

[B67] Tian J.-S. Peng G.-J. Wu Y.-F. Zhou J.-J. Xiang H. Gao X.-X. (2016). A GC-MS Urinary Quantitative Metabolomics Analysis in Depressed Patients Treated with TCM Formula of Xiaoyaosan. J. Chromatogr. B 1026, 227–235. 10.1016/j.jchromb.2015.12.026 26733091

[B68] Wodlej C. Riedl S. Rinner B. Leber R. Drechsler C. Voelker D. R. (2019). Interaction of Two Antitumor Peptides with Membrane Lipids - Influence of Phosphatidylserine and Cholesterol on Specificity for Melanoma Cells. PLoS One 14, e0211187. 10.1371/journal.pone.0211187 30682171 PMC6347193

[B69] Yin P. Zhao X. Li Q. Wang J. Li J. Xu G. (2006). Metabonomics Study of Intestinal Fistulas Based on Ultraperformance Liquid Chromatography Coupled with Q-TOF Mass Spectrometry (UPLC/Q-TOF MS). J. Proteome Res. 5, 2135–2143. 10.1021/pr060256p 16944924

[B70] Yoshida S. Fukumoto S. Kawaguchi H. Sato S. Ueda R. Furukawa K. (2001). Ganglioside G(D2) in Small Cell Lung Cancer Cell Lines: Enhancement of Cell Proliferation and Mediation of Apoptosis. Cancer Res. 61, 4244–4252. 11358851

[B71] Young B. P. Shin J. J. H. Orij R. Chao J. T. Chao S. C. Guan X. L. (2010). Phosphatidic Acid Is a pH Biosensor that Links Membrane Biogenesis to Metabolism. Science 329, 1085–1088. 10.1126/science.1191026 20798321

[B72] Yu S. Wang D. Shao Y. Zhang T. Xie H. Jiang X. (2019). SP1-induced lncRNA TINCR Overexpression Contributes to Colorectal Cancer Progression by Sponging miR-7-5p. Aging 11, 1389–1403. 10.18632/aging.101839 30853664 PMC6428101

[B73] Zalloua P. Kadar H. Hariri E. Abi Farraj L. Brial F. Hedjazi L. (2019). Untargeted Mass Spectrometry Lipidomics Identifies Correlation between Serum Sphingomyelins and Plasma Cholesterol. Lipids Health Dis. 18, 38. 10.1186/s12944-018-0948-5 30711004 PMC6359757

[B74] Zhang F. Zhang Y. Zhao W. Deng K. Wang Z. Yang C. (2017). Metabolomics for Biomarker Discovery in the Diagnosis, Prognosis, Survival and Recurrence of Colorectal Cancer: a Systematic Review. Oncotarget 8, 35460–35472. 10.18632/oncotarget.16727 28389626 PMC5471069

[B75] Zhao K. Zhou H. Zhao X. Wolff D. W. Tu Y. Liu H. (2012). Phosphatidic Acid Mediates the Targeting of tBid to Induce Lysosomal Membrane Permeabilization and Apoptosis. J. Lipid Res. 53, 2102–2114. 10.1194/jlr.M027557 22761256 PMC3435543

[B76] Zheng X. Tsuchiya K. Okamoto R. Iwasaki M. Kano Y. Sakamoto N. (2011). Suppression of Hath1 Gene Expression Directly Regulated by Hes1 via Notch Signaling Is Associated with Goblet Cell Depletion in Ulcerative Colitis. Inflamm. Bowel Dis. 17, 2251–2260. 10.1002/ibd.21611 21987298

