# Supplementary material for: Multi-Omics Prognostic Signatures Based on Lipid Metabolism for Colorectal Cancer
Source: Front Cell Dev Biol. 2022 Feb 11;9:811957. doi: 10.3389/fcell.2021.811957 (PMC8874334; doi:10.3389/fcell.2021.811957)
Supplement: Supplementary file 1 [file DataSheet4.docx]

| **Characteristics** | **Variates** | **Training (TCGA)**  **(n=482)** | **Test (GEO) (n=395)** | | |
| --- | --- | --- | --- | --- | --- |
|  |  |  | **GSE17536** | **GSE38832** | **GSE103480** |
| **Age** | **<= 65** | 226(44.14) | 56(45.53) | / | 55(36.18) |
|  | **> 65** | 286(55.86) | 67(54.47) | / | 96(63.16) |
|  | **unknow** | / | / | / | 1(0.66) |
| **Gender** | **Female** | 232(45.31) | 58(47.15) | / | 68(44.74) |
|  | **Male** | 280(54.69) | 65(52.85) | / | 84(55.26) |
| **T staging** | **Tis/1-2** | 109(21.29) | / | / | 7(4.61) |
|  | **T3-4** | 403(78.71) | / | / | 145(95.39) |
| **N staging** | **N0** | 300(58.60) | / | / | 80(52.63) |
|  | **N1-2** | 211(41.21) | / | / | 72(47.37) |
|  | **NX** | 1(0.19) | / | / | / |
| **M staging** | **M0** | 385(75.20) | / | / | 85(55.92) |
|  | **M1** | 72(14.06) | / | / | / |
|  | **Mx+unknow** | 55(10.74) | / | / | 67(44.08) |
| **Pathological stage** | **Stage Ⅰ-Ⅱ** | 283(55.27) | 81(65.85) | 53(44.17) | 80(52.63) |
|  | **Stage Ⅲ-Ⅳ** | 214(41.80) | 42(34.15) | 67(55.83) | 72(47.37) |
|  | **unknow** | 15(2.93) | / | / | / |

**Table S4: The summary of clinical characteristics of CRC patients in the training and test group**
