# Supplementary material for: Multi-Omics Prognostic Signatures Based on Lipid Metabolism for Colorectal Cancer
Source: Front Cell Dev Biol. 2022 Feb 11;9:811957. doi: 10.3389/fcell.2021.811957 (PMC8874334; doi:10.3389/fcell.2021.811957)
Supplement: Supplementary file 2 [file DataSheet3.docx]

**Table S3: The 342 DEGs of Mebrown**

| **Probes** | **Module Color** |
| --- | --- |
| PIGZ | brown |
| MT1X | brown |
| ISX | brown |
| PLCD1 | brown |
| PAQR5 | brown |
| TDP2 | brown |
| CAPN5 | brown |
| ITM2C | brown |
| SLC51B | brown |
| ITLN1 | brown |
| ENHO | brown |
| DMRTA1 | brown |
| OTOP2 | brown |
| TRPV3 | brown |
| DHRS9 | brown |
| SEMA6D | brown |
| GBA3 | brown |
| BMP3 | brown |
| SRI | brown |
| ANPEP | brown |
| AOC1 | brown |
| CASP5 | brown |
| CDC25B | brown |
| GCNT3 | brown |
| FOXQ1 | brown |
| ARHGAP44 | brown |
| MARVELD3 | brown |
| MYPN | brown |
| ABCG2 | brown |
| TMEM220 | brown |
| UGT2B15 | brown |
| GUCA2A | brown |
| C15orf48 | brown |
| CA2 | brown |
| CEACAM7 | brown |
| CPT2 | brown |
| DNASE1L3 | brown |
| SLC4A4 | brown |
| RPL10L | brown |
| SLC13A1 | brown |
| SGK2 | brown |
| CDKN2B-AS1 | brown |
| HSD17B2 | brown |
| DRD5 | brown |
| ABCC13 | brown |
| KRT80 | brown |
| VSIG2 | brown |
| G6PC | brown |
| GGT6 | brown |
| PHLPP2 | brown |
| LGALS4 | brown |
| CNTFR | brown |
| CNNM4 | brown |
| TUBAL3 | brown |
| HSD11B2 | brown |
| ACOX1 | brown |
| NR3C2 | brown |
| DPF3 | brown |
| CNNM2 | brown |
| KRT20 | brown |
| GDPD3 | brown |
| FUCA1 | brown |
| NXPE1 | brown |
| CHGA | brown |
| TRIM40 | brown |
| XDH | brown |
| LINC01351 | brown |
| SCNN1B | brown |
| SLC44A4 | brown |
| ATP13A4 | brown |
| GSTA1 | brown |
| TTC22 | brown |
| TRPM6 | brown |
| ZNF575 | brown |
| STYK1 | brown |
| SULT1A1 | brown |
| SST | brown |
| MUC4 | brown |
| RNF152 | brown |
| B3GALT5 | brown |
| PXMP2 | brown |
| KCNE2 | brown |
| LINC00955 | brown |
| ALPI | brown |
| MADCAM1 | brown |
| BCAS1 | brown |
| PRSS22 | brown |
| ASPG | brown |
| RDH5 | brown |
| TTLL6 | brown |
| CES2 | brown |
| ACKR2 | brown |
| CHP1 | brown |
| BMP2 | brown |
| HPGD | brown |
| CDHR2 | brown |
| LIPH | brown |
| DHRS7C | brown |
| ZG16 | brown |
| FABP1 | brown |
| MGAM | brown |
| LRRC66 | brown |
| DISP2 | brown |
| SLC9A3 | brown |
| DHRS11 | brown |
| TRIB3 | brown |
| NPY6R | brown |
| SI | brown |
| PLAC8 | brown |
| SLC9A1 | brown |
| ST6GALNAC6 | brown |
| SPINK2 | brown |
| SLC5A11 | brown |
| MEP1A | brown |
| RHOF | brown |
| PTGDR | brown |
| ABCB11 | brown |
| MALL | brown |
| CLMN | brown |
| LINC01133 | brown |
| SLC52A1 | brown |
| MT1E | brown |
| HHLA2 | brown |
| HAGLR | brown |
| ETV4 | brown |
| CLDN23 | brown |
| NAAA | brown |
| TM6SF2 | brown |
| PDE6A | brown |
| ENDOD1 | brown |
| TINCR | brown |
| FAM151A | brown |
| SEMA6A | brown |
| HS3ST6 | brown |
| MUC2 | brown |
| TRIM29 | brown |
| TLCD2 | brown |
| MT1G | brown |
| TSPAN1 | brown |
| UGT2A3 | brown |
| PGM1 | brown |
| SLC16A9 | brown |
| P2RY1 | brown |
| CES3 | brown |
| MT2A | brown |
| C10orf99 | brown |
| DUSP21 | brown |
| GUCA2B | brown |
| BEST2 | brown |
| SLC25A20 | brown |
| LAMA1 | brown |
| EDN3 | brown |
| KLF4 | brown |
| PCSK5 | brown |
| SLC51A | brown |
| NAALADL1 | brown |
| SLC6A19 | brown |
| ENAM | brown |
| CAPN9 | brown |
| TMEM171 | brown |
| TEX11 | brown |
| VSTM2A | brown |
| FCGBP | brown |
| SLC13A2 | brown |
| SMOX | brown |
| PCK1 | brown |
| SLC17A1 | brown |
| SLCO4A1 | brown |
| PDX1 | brown |
| CLDN8 | brown |
| WDR78 | brown |
| HSD3B2 | brown |
| B3GNT7 | brown |
| ACADS | brown |
| BTNL3 | brown |
| NFE2L3 | brown |
| TMEM253 | brown |
| CCL28 | brown |
| FMO5 | brown |
| C4orf19 | brown |
| AKR1B10 | brown |
| ENPP6 | brown |
| MS4A12 | brown |
| CHAD | brown |
| SLC4A10 | brown |
| DSC2 | brown |
| SLC26A2 | brown |
| MOB3B | brown |
| PCAT18 | brown |
| SIAE | brown |
| MGLL | brown |
| LINC00507 | brown |
| CLDN1 | brown |
| ACVRL1 | brown |
| MT1F | brown |
| PYY | brown |
| TMEM37 | brown |
| ZBTB7B | brown |
| PLCE1 | brown |
| NR1H4 | brown |
| ATOH1 | brown |
| SLC17A4 | brown |
| LYPD8 | brown |
| SLC26A3 | brown |
| PTPRH | brown |
| TMEM72 | brown |
| CEACAM1 | brown |
| UGDH | brown |
| APOBR | brown |
| PDZD3 | brown |
| SMPD3 | brown |
| EPB41L4B | brown |
| GPR15 | brown |
| RETSAT | brown |
| HRCT1 | brown |
| CASP7 | brown |
| SCIN | brown |
| CD177 | brown |
| PBLD | brown |
| MB | brown |
| PTGDR2 | brown |
| LGALS2 | brown |
| B3GALT1 | brown |
| SMIM6 | brown |
| ABHD3 | brown |
| SLC36A1 | brown |
| FEV | brown |
| BLACAT1 | brown |
| P2RY4 | brown |
| PAPSS2 | brown |
| SLC1A1 | brown |
| C11orf86 | brown |
| SLC17A8 | brown |
| SLC22A18AS | brown |
| TNFRSF12A | brown |
| INSL5 | brown |
| CHST5 | brown |
| PKIB | brown |
| SLC22A5 | brown |
| CA7 | brown |
| NEU4 | brown |
| TPSG1 | brown |
| DAO | brown |
| CA1 | brown |
| RHBDL2 | brown |
| GPT | brown |
| ADTRP | brown |
| BTNL8 | brown |
| MT1M | brown |
| SPIB | brown |
| ADH1C | brown |
| FABP2 | brown |
| HOXD1 | brown |
| MEP1B | brown |
| SLCO4C1 | brown |
| LRRC19 | brown |
| SLC35D1 | brown |
| CHP2 | brown |
| DHDDS | brown |
| STBD1 | brown |
| PPP1R14D | brown |
| SLC23A1 | brown |
| APPL2 | brown |
| NXPE4 | brown |
| NR5A2 | brown |
| SMPDL3A | brown |
| CDHR5 | brown |
| EDN2 | brown |
| AMPD1 | brown |
| ATP8B1 | brown |
| HAPLN1 | brown |
| CD163L1 | brown |
| CA4 | brown |
| NEURL1 | brown |
| IGSF9 | brown |
| ETHE1 | brown |
| S100A11 | brown |
| MYH15 | brown |
| SLC9A2 | brown |
| B4GALNT2 | brown |
| SLC41A2 | brown |
| TGFBI | brown |
| VILL | brown |
| P2RX4 | brown |
| CYP2B7P | brown |
| PADI2 | brown |
| ENTPD5 | brown |
| KHDRBS2 | brown |
| KRT9 | brown |
| PTGER4 | brown |
| USP2 | brown |
| TAT | brown |
| CWH43 | brown |
| HTR4 | brown |
| MAOA | brown |
| VIPR1 | brown |
| C2orf88 | brown |
| BCAR3 | brown |
| CREB3L3 | brown |
| RILP | brown |
| C1orf210 | brown |
| C1orf115 | brown |
| LDHD | brown |
| AQP8 | brown |
| SCGB2A1 | brown |
| CLCA1 | brown |
| MOGAT2 | brown |
| CA12 | brown |
| GCG | brown |
| CDKN2B | brown |
| CLCA4 | brown |
| ST6GALNAC1 | brown |
| SLC22A23 | brown |
| BARX2 | brown |
| BRINP3 | brown |
| TPH1 | brown |
| GCNT2 | brown |
| LIMA1 | brown |
| IQGAP2 | brown |
| MYO1A | brown |
| CFD | brown |
| GDPD2 | brown |
| BEST4 | brown |
| SULT1B1 | brown |
| B3GNT6 | brown |
| GPD1L | brown |
| CDH3 | brown |
| SLC25A34 | brown |
| GNA11 | brown |
| PAG1 | brown |
| TMCC3 | brown |
| MMP28 | brown |
| ARL14 | brown |
| GPA33 | brown |
| AHCYL2 | brown |
| PRKG2 | brown |
| ETFDH | brown |
| SLC30A10 | brown |
| RBM47 | brown |
| PEX26 | brown |
| CCDC152 | brown |
| HEPACAM2 | brown |
