# Supplementary figures and images for: Multi-Omics Prognostic Signatures Based on Lipid Metabolism for Colorectal Cancer
Source: Front Cell Dev Biol. 2022 Feb 11;9:811957. doi: 10.3389/fcell.2021.811957 (PMC8874334; doi:10.3389/fcell.2021.811957)

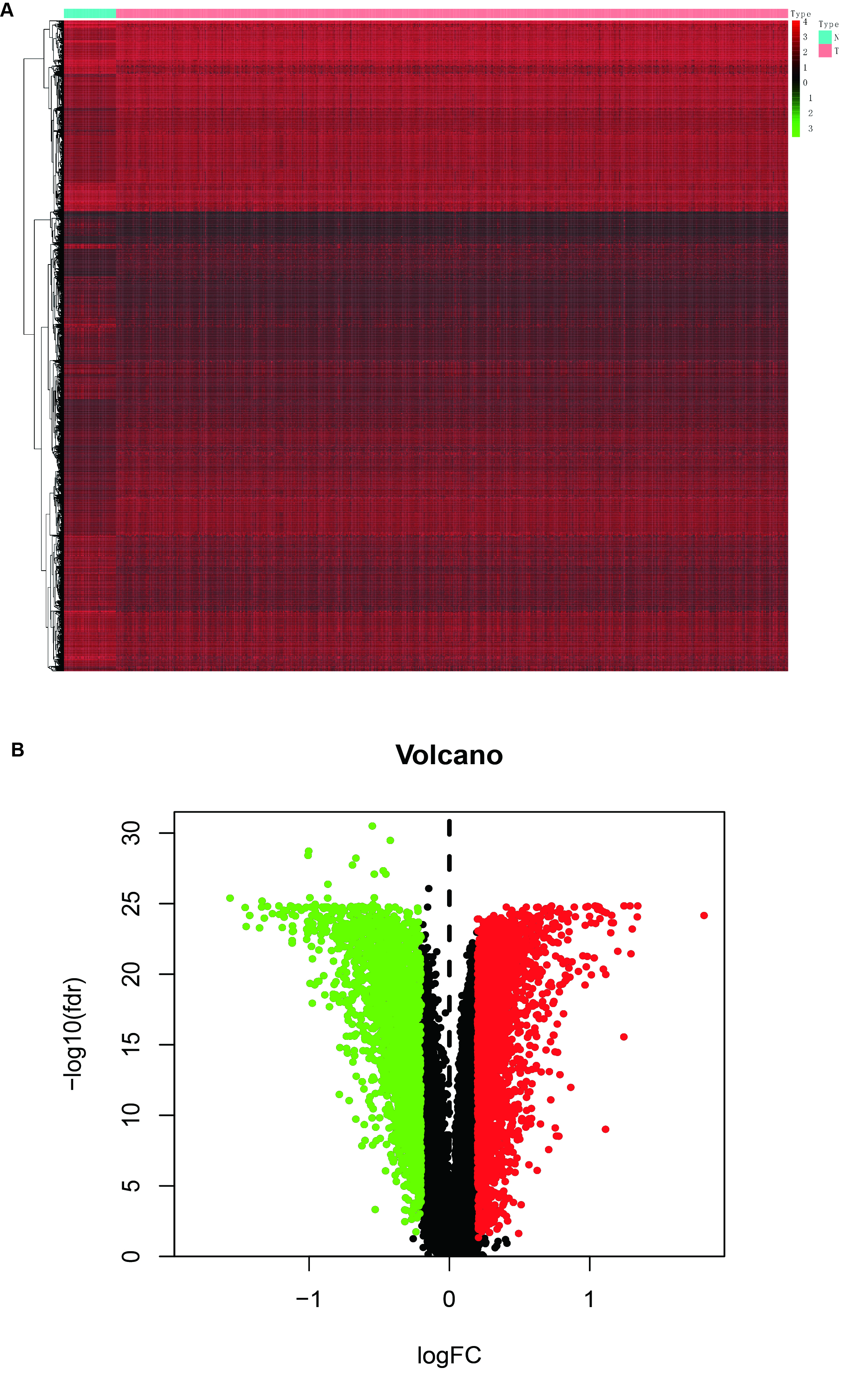

Supplement: Supplementary file 3 [file Image1.JPEG]

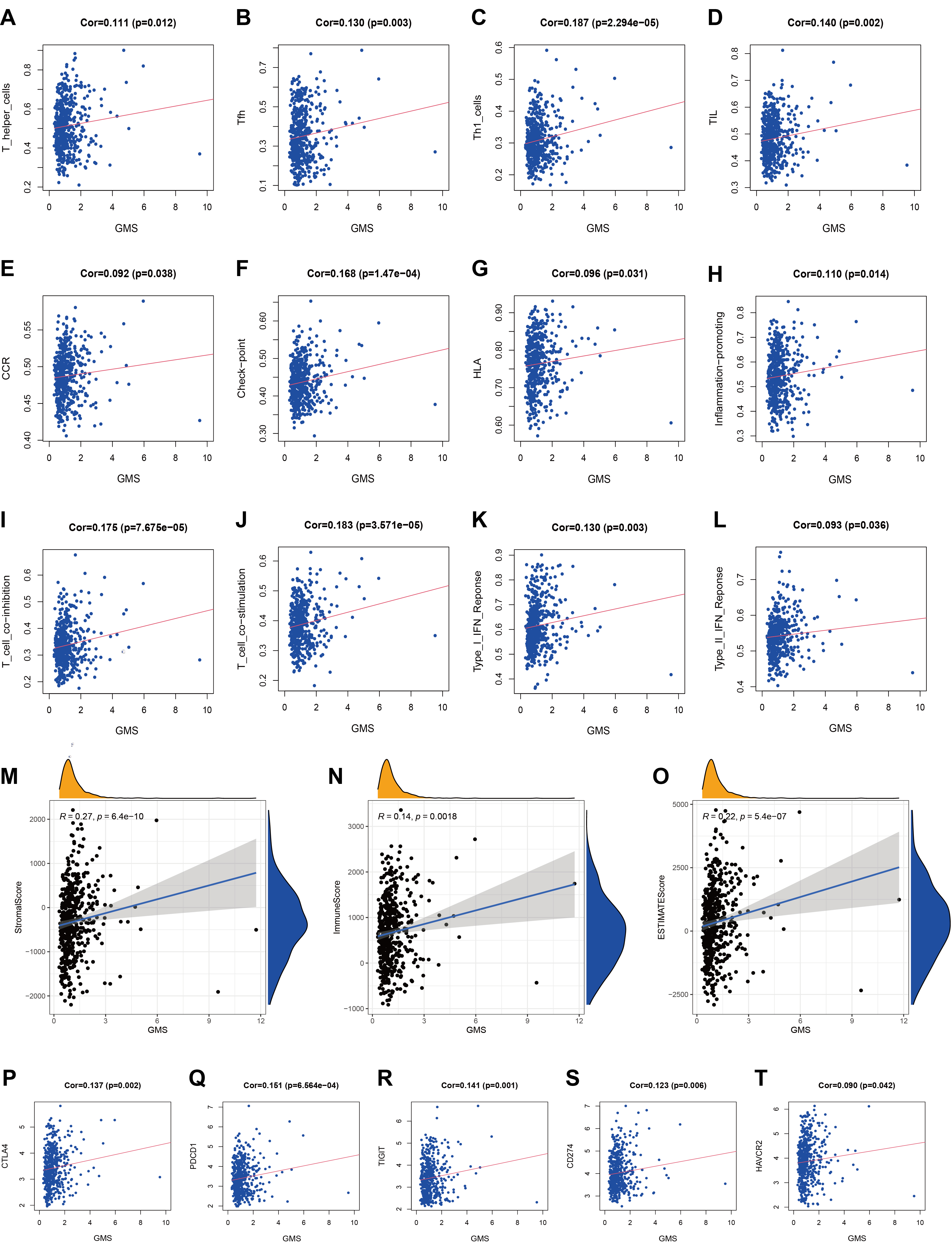

Supplement: Supplementary file 4 [file Image2.JPEG]
