# Supplementary material for: Multi-Omics Prognostic Signatures Based on Lipid Metabolism for Colorectal Cancer
Source: Front Cell Dev Biol. 2022 Feb 11;9:811957. doi: 10.3389/fcell.2021.811957 (PMC8874334; doi:10.3389/fcell.2021.811957)
Supplement: Supplementary file 5 [file DataSheet1.docx]

**Table S1: The grouping statistics of Kgroup1 and Kgroup2**

| **Characteristics** | **Variates** | **K-group1(Percentage)** | **K-group2(Percentage)** |
| --- | --- | --- | --- |
| **Age** | **<= 65** | 44 (61.97%) | 124 (75.15%) |
|  | **> 65** | 27 (38.03%) | 41 (24.85%) |
| **Gender** | **Female** | 28 (39.44%) | 78 (47.27%) |
|  | **Male** | 43 (60.56%) | 87 (52.73%) |
| **Histological type** | **Adenocarcinoma** | 41 (57.75%) | 150 (90.91%) |
|  | **Mucinous adenocarcinoma** | 30 (42.25%) | 15 (9.09%) |
| **T staging** | **T1-2** | 5 (7.04%) | 30 (18.18%) |
|  | **T3-4** | 66 (92.96%) | 135 (81.82%) |
| **N staging** | **N0** | 29 (40.85%) | 95 (57.58%) |
|  | **N1-2** | 42 (59.15%) | 70 (42.42%) |
| **M staging** | **M0** | 63 (88.73%) | 162 (98.18%) |
|  | **M1** | 8 (11.27%) | 3 (1.82%) |
| **Pathological stage** | **Stage Ⅰ-Ⅱ** | 28 (39.47%) | 96 (58.18%) |
|  | **Stage Ⅲ-Ⅳ** | 43 (60.56%) | 69 (41.82) |
